# The ERK signaling target RNF126 regulates anoikis resistance in cancer cells by changing the mitochondrial metabolic flux

**DOI:** 10.1038/celldisc.2016.19

**Published:** 2016-07-26

**Authors:** Seiko Yoshino, Toshiro Hara, Hiroki J Nakaoka, Akane Kanamori, Yoshinori Murakami, Motoharu Seiki, Takeharu Sakamoto

**Affiliations:** 1 Division of Cancer Cell Research, Institute of Medical Science, The University of Tokyo, Tokyo, Japan; 2 Division of Molecular Pathology, Institute of Medical Science, The University of Tokyo, Tokyo, Japan; 3 Faculty of Medicine, Institute of Medical, Pharmaceutical and Health Sciences, Kanazawa University, Kanazawa, Japan

**Keywords:** anoikis, cancer, metabolism, PDK, RNF126, ubiquitination

## Abstract

Loss of anchorage to the extracellular matrix leads to apoptosis (anoikis) in normal cells, but cancerous cells are usually resistant to such stress. Here we report the pivotal role of an E3 ubiquitin ligase, ring-finger protein 126 (RNF126), in the resistance of cancer cells to the stress associated with non-adherent conditions. Non-adherent cancer cells exhibited increased flux through the tricarboxylic acid cycle via increased conversion of pyruvate to acetyl-CoA. RNF126 was found to act as a ubiquitin ligase for pyruvate dehydrogenase kinases (PDKs), resulting in their proteasomal degradation. This decrease in PDK levels allowed pyruvate dehydrogenases to catalyze the conversion of pyruvate to acetyl-CoA. Moreover, depletion of RNF126 or increased expression of PDK1 in cancer cells suppressed colony formation in soft agar as well as tumorigenicity in mice. RNF126 expression in cancer cells was found to be under the control of the extracellular signal-regulated kinase signaling pathway, which is essential for anoikis resistance. Thus, RNF126 is an attractive molecule for treating cancer by selectively targeting anchorage-independent growth.

## Introduction

In addition to cell–cell adhesion, cell–extracellular matrix (ECM) adhesion is also important for tissues and regulates cell growth and differentiation in a strict manner to maintain tissue integrity. Only a few cell types, such as blood cells, do not require cell–ECM adhesion for their survival and growth. Cells that lack adhesion to the ECM usually cease proliferating and undergo cell death, also known as anoikis [1]. Anoikis hass important roles in organ formation during the development in multicellular organisms. For example, during the morphogenesis of mammary acini, detachment of mammary epithelial cells from the ECM leads to the loss of β1-integrin and epidermal growth factor receptor expression, decreased extracellular signal-regulated kinase (ERK) signaling and stabilization of proapoptotic BIM proteins, resulting in apoptosis [2]. Anoikis is another mechanism by which the survival and growth of aberrant cells, after their detachment from the tissue of origin, are prevented. For example, cancer cells disseminated from their primary location can invade surrounding tissue and form distant metastases; anoikis prevents the persistence of such cells. Therefore, invasive and metastatic cancer cells usually acquire anoikis resistance and thereby reduce their dependency on cell–ECM adhesion; these cells can grow in suspension and metastasize from the primary tumor [2–5]. As anoikis resistance is unnecessary for the maintenance of organs by normal cells, this phenomenon is characteristic of malignant cancer cells and therefore constitutes a possible therapeutic target. However, the detailed mechanisms of anoikis resistance are not fully understood and anoikis resistance-targeting drugs are not currently available.

Recently, metabolic deviation of cancer cells from normal cells has attracted attention, and it is becoming increasingly clear that metabolic changes have important roles in the gain and maintenance of cancer traits, including anoikis resistance [2, 6–9]. For example, it has become evident that cells cultured in suspension undergo changes in metabolic flux, from glycolysis to the tricarboxylic acid (TCA) cycle, and in these cells, ATP is produced via the mitochondrial TCA cycle and oxidative phosphorylation (OXPHOS). This metabolic shift has an important role in the anoikis resistance of cancer cells and is regulated by cell growth signaling pathways, such as the ERBB2–ERK pathway [10, 11]. In addition, phosphoglycerate dehydrogenase, which diverts glycolytic carbon to serine and glycine metabolism, is overexpressed in melanoma and some types of breast cancer, and ectopic expression of phosphoglycerate dehydrogenase confers MCF-10A mammary epithelial cells with the ability to resist anoikis [12].

In this study, we demonstrate that ring-finger protein 126 (RNF126), an E3 ubiquitin ligase associated with cell growth [13], has different effects on the growth of attached and suspended cells. Metabolomic analyses revealed that RNF126 expression shifts the metabolic flux to the TCA cycle. In contrast, RNF126 depletion profoundly affected cells grown in suspension, but had little effect on attached cells, whose growth did not depend on the flux change to the TCA cycle. RNF126 regulates conversion of pyruvate to acetyl-CoA, the step that functions as a gatekeeper between glycolysis and the TCA cycle. RNF126 exhibits ubiquitin ligase activity against pyruvate dehydrogenase kinases (PDKs) and negatively regulates the levels of these proteins. Depletion of RNF126 also attenuated tumor growth in mice in a PDK-dependent manner. Thus, RNF126 was identified here as a new regulator of anchorage-independent growth for cancer cells and therefore is a potential target for developing novel cancer therapeutics.

## Results

### RNF126 regulated cell growth selectively under non-adherent conditions

Cancer cells usually acquire an ability to grow in an anchorage-independent manner, which is evaluated by colony-formation assays in soft agar. While comparing the effects of genes under two different culture conditions (culture in dishes and in soft agar), we noticed that knockdown (KD) of a growth-related gene *RNF126* affected cell growth differentially between the two conditions.

**Figure 1:**
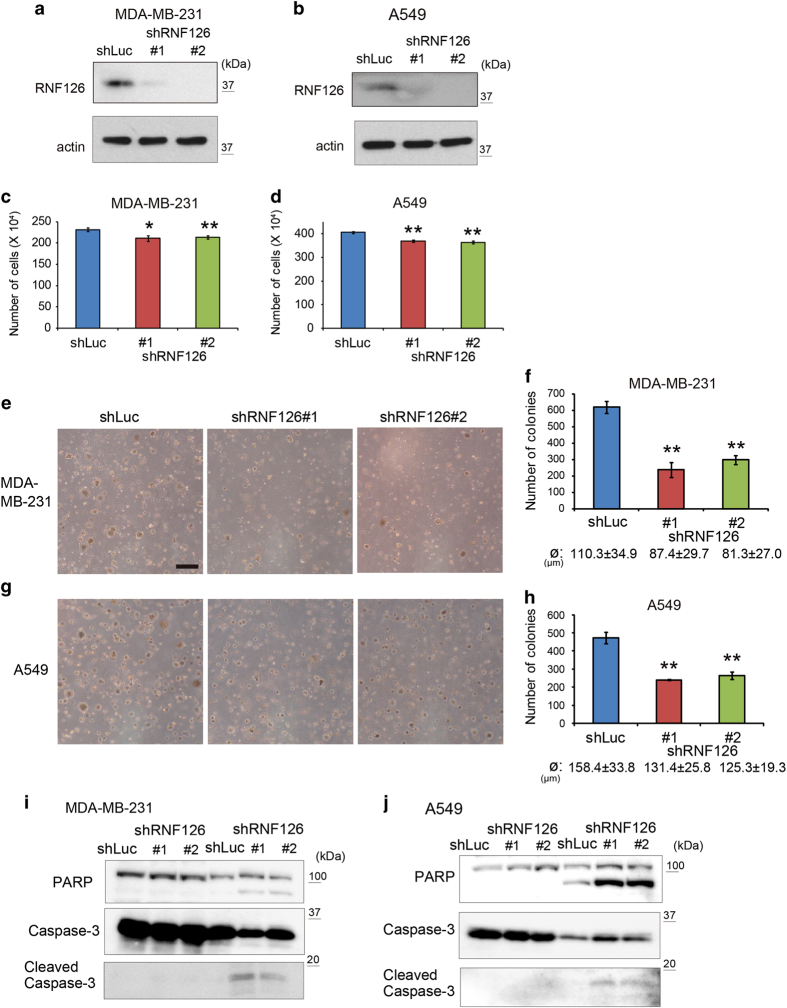
RNF126 promotes the colony-forming capacity of cancer cells in soft agar. (**a** and **b**) Western blot analysis of RNF126 expression in MDA-MB-231 (**a**) and A549 cells (**b**), following RNF126  KD. (**c** and **d**) Growth of RNF126-depleted MDA-MB-231 (**c**) and A549 (**d**) cells in culture dishes. (**e** and **g**) Representative photographs of colony formation in soft agar in control and RNF126-depleted MDA-MB-231 (**e**) and A549 cells (**g**). Bar=500 μm. (**f** and **h**) Number of colonies formed in soft agar by control and RNF126-depleted MDA-MD-231 (**f**) and A549 cells (**h**). The diameter (ø) of the counted colonies is indicated as the average±s.d. (**i** and **j**) Western blot analysis of poly(ADP-ribose) polymerase (PARP), caspase-3 and cleaved caspase-3 in control and RNF126-depleted MDA-MB-231 (**i**) and A549 cells (**j**) that were cultured under attached or detached conditions for 96 h. In (**c**, **d**, **f** and **h**), the error bars indicate the s.d. (*n*=3). The data were analyzed by a *t*-test. *P<0.05; **P<0.01. The data shown in (**a**–**j**) are representative of three independent experiments with similar results.

Previously, we showed that RNF126-KD using short hairpin RNAs (shRNAs) suppressed cell growth significantly [13], suggesting its role as a growth-regulatory gene. Here we depleted RNF126 expression in human MDA-MB-231 breast carcinoma cells and human A549 lung carcinoma cells using two shRNA sequences (shRNF126 #1 and 2), with an shRNA against luciferase mRNA (shLuc) serving as a control (Figure 1a and b). Depletion of RNF126 indeed retarded cell growth significantly, but the effect was only marginal (Figure 1c and d). However, marked difference was observed when cells were seeded into soft agar and the number of colonies formed was counted after 21 days of culture: RNF126 depletion decreased the number and size of colonies formed (Figure 1e–h). Then, we examined whether RNF126 has a role in anoikis resistance in cancer cells. Control and RNF126-depleted MDA-MB-231 and A549 cells were grown for 96 h in standard culture dishes (i.e., in the attached state) or in ultra-low attachment dishes (i.e., in the detached state) and analyzed in trypan blue-exclusion assays. When cells were cultured in the detached state, RNF126 depletion increased the percentage of trypan blue-positive cells, indicating that RNF126 is necessary for cancer cells to survive in the detached state (Supplementary Figure S1). Lysates from these cells were also subjected to immunoblotting to detect apoptosis-associated cleavages of poly(ADP-ribose) polymerase and caspase-3. RNF126 depletion increased poly(ADP-ribose) polymerase and caspase-3 cleavage in the lysates from detached cells (Figure 1i and j). Thus, RNF126 has a key role in anoikis resistance in cancer cells.

### Effect of RNF126 on tumor growth in mice

**Figure 2:**
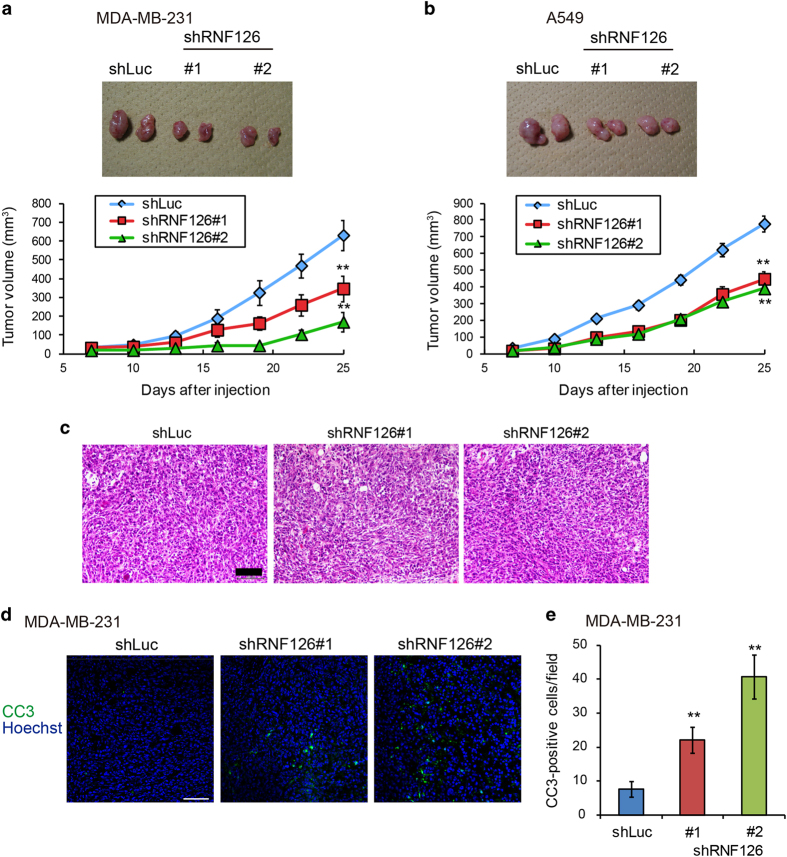
RNF126 promotes tumorigenicity. (**a** and **b**) Representative photographs (upper panel; day 25) and rate of growth (lower panel) following subcutaneous implantation of control and RNF126-depleted MDA-MB-231 (**a**) and A549 cells (**b**) in immunodeficient mice. (**c**) Hematoxylin and eosin staining of tumor tissues derived from control and RNF126-depleted MDA-MB-231 cells at day 25. Scale bar=100 μm. (**d**) Immunostaining of cleaved caspase-3 (CC3) in tumor tissues of control and RNF126-depleted MDA-MB-231 cells at day 25. Bar=200 μm. (**e**) CC3-positive cells in tumor sections in (**d**) were counted. In (**a**, **b** and **e**), the error bars indicate the s.e.m.; *n*=8 from two independent experiments (*n*=4 and *n*=4, respectively); the data shown were analyzed using the Mann–Whitney *U*-test. **P<0.01.

As anoikis resistance is important for tumorigenic activity of cancer cells *in vivo*, we assessed the effect of RNF126 on tumor formation. RNF126-depleted MDA-MB-231 cells were implanted subcutaneously into immunodeficient mice and tumor growth was monitored. RNF126-depleted cells formed tumors that were 50–70% smaller than those formed by the control cells (Figure 2a). A similar effect was observed with A549 cells, as RNF126-KD decreased tumor volume by ~40% (Figure 2b). Thus, RNF126 depletion had a greater impact on colony formation *in vitro* and tumor formation *in vivo* than in cultured monolayers *in vitro*. Next, we analyzed the histology of MDA-MB-231 tumors. Both control and RNF126-depleted tumors derived from MDA-MB-231 cells showed solid nests of large epithelial cells with an abundant cytoplasm and prominent nucleoli, and no apparent difference was discerned among them (Figure 2c). However, we observed an increased number of apoptotic cells comprising RNF126-depleted MDA-MB-231 cells, as compared with the control cells (Figure 2d and e), suggesting that RNF126 contributes to anoikis resistance *in vivo*.

### ERK signaling promoted RNF126 expression

**Figure 3:**
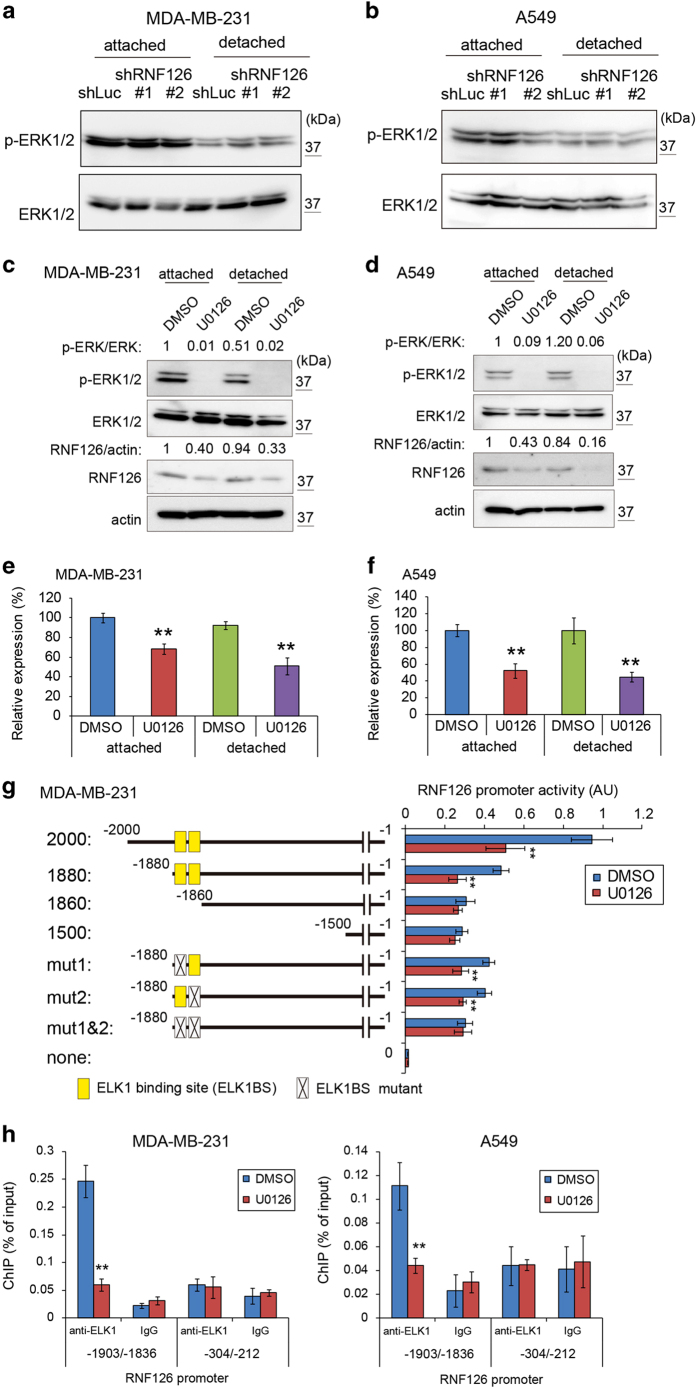
The ERK signaling pathway promotes RNF126 expression. (**a** and **b**) Western blot analysis of ERK1/2 and phosphorylated (p)-ERK1/2 in control and RNF126-depleted MDA-MB-231 (**a**) and A549 cells (**b**). (**c** and **d**) Western blot analysis of RNF126, ERK1/2 and phosphorylated-ERK1/2 in vehicle (dimethyl sulfoxide (DMSO))- or MEK inhibitor U0126- (10 μm) treated MDA-MB-231 (**c**) and A549 cells (**d**). Results of the densitometric analysis of bands in western blots are presented. (**e** and **f**) mRNA levels of *RNF126* in vehicle (DMSO)- or MEK inhibitor U0126- (10 μm) treated MDA-MB-231 (**e**) and A549 cells (**f**) were analyzed by real-time PCR. Error bars indicate the s.d. (*n*=3). The data were analyzed by a *t*-test. **P<0.01. (**g**) RNF126 promoter activity was analyzed in MDA-MB-231 cells treated with or without U0126 (10 μm). Error bars indicate the s.d. (*n*=3). The data were analyzed by a *t*-test. **P<0.01. (**h**) ChIP real-time PCR of the RNF126 promoter using anti-ELK-1 or control immunoglobulin G (IgG) in MDA-MB-231 cells (left) and A549 cells (right) treated with or without U0126 (10 μm). Error bars indicate the s.d. (*n*=3). The data were analyzed by a *t*-test. **P<0.01. The data shown in (**a**–**h**) are representative of three independent experiments with similar results.

Detachment of cells from the ECM leads to decreased ERK signaling, and activation of this pathway causes anoikis resistance [2]. Thus, we examined whether RNF126 affects the ERK signaling pathway. RNF126 depletion in MDA-MB231 and A549 cells did not affect the phosphorylation status of ERK1/2 when cells were grown in standard culture dishes (i.e., the attached state) or in ultra-low attachment dishes (i.e., the detached state) (Figure 3a and b). We then examined whether RNF126 expression was regulated by the ERK signaling pathway, using the MEK inhibitor U0126. U0126 treatment decreased RNF126 levels in MDA-MB-231 and A549 cells in both the attached and detached states (Figure 3c and d). RNF126 mRNA levels were also decreased by U0126 treatment (Figure 3e and f). These data indicated that RNF126 is a downstream target of the ERK signaling pathway. Although the phosphorylation levels of ERK1/2 decreased to ~50% in MDA-MB-231 cells under the detached condition, the ERK1/2 phosphorylation levels appeared to be sufficient to maintain RNF126 expression (Supplementary Figure S2). Next, we examined whether the promoter activity of RNF126 is regulated by the ERK signaling pathway in reporter assays. The reporter activities of 2 000 and 1 880 bp RNF126 promoter fragments were decreased by U0126 treatment to ~50% of that observed following vehicle-control treatment, whereas those of 1 860 and 1 500 bp fragments did not decrease (Figure 3g). ELK1 is a target transcriptional factor of ERK, and we identified two tandem ELK1 binding sites (ELK1BSs) between nucleotide positions −1 880 and −1 860 in the RNF126 promoter region using the JASPAR database (http://jaspardev.genereg.net/) (Supplementary Table S1). The core sequence of ELK1BSs is 5′-CCGGAA-3′, where the underlined GA nucleotides are essential for the binding activity. Thus, we prepared modified 1 880-bp RNF126 promoter fragments, where GA was replaced with AG in one or both ELK1BSs (Figure 3g). Each ELK1BS mutation between nucleotides −1 880 and −1 860 of the RNF126 promoter showed a decreased, but significant response to U0126 treatment in reporter assays (Figure 3g; mut1 and mut2). However, mutations in both ELK1BSs of the RNF126 promoter abolished responses to U0126 treatment (Figure 3g; mut1 and mut2). In parallel with the reporter assay, a chromatin immunoprecipitation (ChIP) assay using an anti-ELK1 antibody showed that U0126 treatment diminished ELK1 binding to an RNF126 promoter fragment containing both ELK1BSs (nucleotides −1 903 to −1 836). In contrast, ELK1 binding to another RNF126 promoter fragment containing two potential ELK1BSs (Supplementary Table S1; nucleotides −304 to −212) was not reduced in MDA-MB-231 and A549 cells (Figure 3h). Thus, ERK signaling pathway regulates RNF126 promoter activity via ELK1.

### RNF126 regulated metabolic flux from glycolysis to the TCA cycle

**Figure 4:**
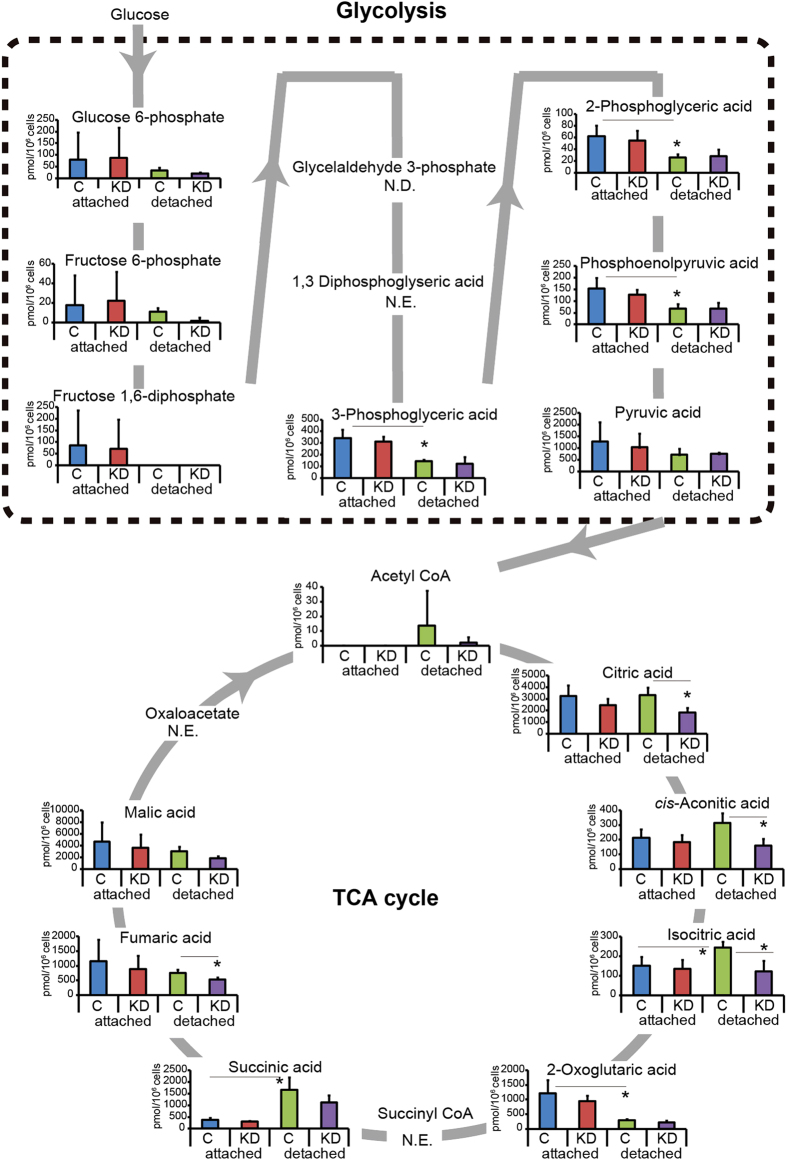
RNF126 depletion causes metabolic changes. Capillary electrophoresis-time of flight/mass spectrometry (CE-TOF/MS) analysis of glycolytic and TCA cycle metabolites in control (C) and RNF126-depleted (KD) MDA-MB-231 cells that had been cultured in attached or detached states from three independent experiments. Error bars indicate the s.d. (*n*=3). The data were analyzed using a *t*-test. *P<0.05. ND, not detected; NE, not examined.

Recently, the adherence or non-adherence of cells to the ECM was reported to affect metabolic flux from glycolysis to the TCA cycle; increased metabolic flux to the TCA cycle has also been found to cause anoikis resistance [10, 11]. We therefore analyzed the effect of RNF126 expression on glycolysis and the TCA cycle. MDA-MB-231 cells were grown in normal culture dishes (attached state) or in ultra-low attachment dishes (detached state), and representative metabolites from both pathways were analyzed by mass spectrometry (Figure 4). The levels of these metabolites did not differ significantly between the control and RNF126-depleted cells when cells were grown in conventional culture dishes (Figure 4, attached state). However, RNF126 depletion significantly affected the levels of TCA cycle metabolites in detached cells. Under the detached condition, three metabolites of glycolysis (3-phosphoglyceric acid, 2-phosphoglyceric acid and phosphoenolpyruvic acid) decreased, whereas two metabolites of the TCA cycle (isocitric acid and succinic acid) increased and one metabolite (2-oxoglutaric acid) of the TCA cycle decreased significantly in MDA-MB-231 cells. Thus, the metabolic status of the cells differed, depending on the cellular attachment to the ECM. RNF126 depletion reduced the accumulation of citric acid, *cis*-aconitic acid, isocitric acid and fumaric acid in the detached cells (Figure 4, detached state).

**Figure 5:**
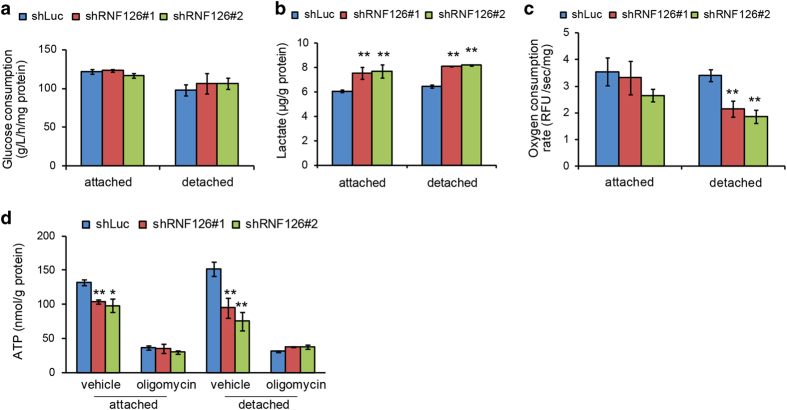
RNF126 depletion caused an OXPHOS defect. (**a–d**) Control and RNF126-depleted MDA-MB-231 cells were cultured under attached or detached conditions, after which they were subjected to assays to determine glucose consumption (**a**) Lactate levels in the culture media (**b**), oxygen consumption (**c**) and cellular ATP contents (**d**). In (**a–d**), error bars indicate the s.d. (*n*=3). The data were analyzed using a *t*-test. *P<0.05; **P<0.01. The data shown are representative of three independent experiments with similar results.

Because RNF126 appeared to decrease metabolic flow through the TCA cycle, we analyzed the effects of RNF126 on upstream glycolysis steps by measuring glucose consumption and lactate production. Glucose consumption was not significantly affected by RNF126 depletion in either attached or detached cells (Figure 5a). In contrast, lactate production was increased by RNF126 depletion in both attached and detached cells (Figure 5b), suggesting that depletion of RNF126 prevents pyruvate from entering the TCA cycle.

If RNF126 regulates the metabolic flow of pyruvate to the TCA cycle, it would affect the oxygen consumption rate and ATP production by OXPHOS. Indeed, oxygen consumption was significantly decreased in RNF126-depleted cells, compared with that observed in control cells (Figure 5c). The effect was more marked in detached cells than in attached cells, potentially reflecting the increased metabolic flow in the TCA cycle (Figure 4).

A similar effect was observed in terms of cellular ATP production (Figure 5d). Because oligomycin specifically inhibits ATP production via OXPHOS, but not via glycolysis, we treated the cells with oligomycin and then measured their ATP levels. Oligomycin suppressed the ATP levels by 70–80% of those observed in non-treated cells and abolished the effect of RNF126 depletion almost completely (Figure 5d). Because oligomycin-resistant ATP production was unaffected by RNF126 depletion, it appeared that RNF126 does not have a role in ATP production by glycolysis. Taken together, these data suggested that RNF126 promotes pyruvate influx into the TCA cycle and that the effect of RNF126 was more pronounced in detached cells.

### RNF126 regulated PDK protein levels

**Figure 6:**
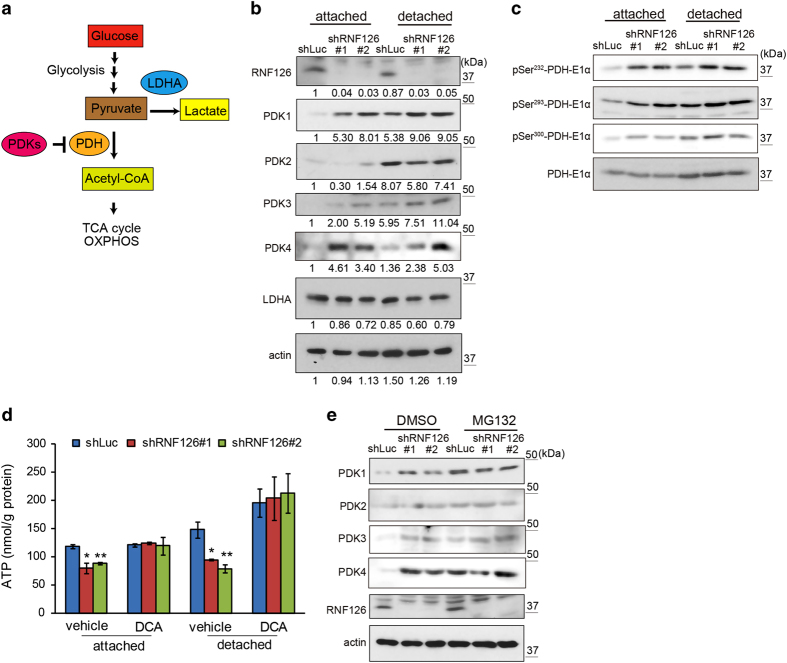
RNF126 regulates the protein-expression levels of PDK1, PDK3 and PDK4. (**a**) Schematic illustration of pyruvate conversion to lactate or acetyl-CoA. (**b**) Western blot analysis of PDKs and LDHA expression in control and RNF126-depleted MDA-MB-231 cells cultured in the attached or detached state. Results of the densitometric analysis of bands in western blots are presented. (**c**) Western blot analysis of PDH-E1α and its phosphorylation levels (pSer^232^, pSer^293^ and pSer^300^) in control and RNF126-depleted cells cultured in the attached or detached state. (**d**) The PDK inhibitor DCA restored ATP levels in RNF126-depleted cells. Error bars indicate the s.d. (*n*=3). The data were analyzed using a *t*-test. *P<0.05; **P<0.01. (**e**) Exposure to the proteasomal inhibitor MG132 eliminated differences in the levels of the PDK1, PDK3 and PDK4 proteins between control and RNF126-KD cells. The data shown in (**b**–**e**) are representative of three independent experiments with similar results.

Pyruvate is converted to acetyl-CoA by PDH complexes (Figure 6a) [14, 15]. The enzymatic activity of PDH complexes can be inhibited by phosphorylation of their E1α subunits via the activities of PDKs (Figure 6a). Among the four members of the PDK family, PDK1 expression can be induced by hypoxia-inducible factor-1 during hypoxia [16, 17]. Expression of PDK4 is reported to increase upon detachment of cells from the ECM, and its expression can be inhibited by ERK-dependent signaling in cancer cells [10]. Because RNF126 appeared to promote the flux of pyruvate into the TCA cycle, we analyzed the expression levels of PDKs and RNF126 in MDA-MB-231 cells by western blotting (Figure 6b). The expression levels of PDKs 1–4 were lower in attached cells compared with those in detached cells, and RNF126 was expressed in both attached and detached cells at similar levels (Figure 6b, shLuc columns). Interestingly, RNF126 depletion in the attached cells increased the protein-expression levels of PDK1, PDK3 and PDK4, but not that of PDK2 (Figure 6b, shRNF126 #1 and 2). A similar effect of RNF126 depletion on PDKs was observed in detached cells. Protein levels of lactate dehydrogenase A (LDHA) were similar between attached and detached cells, and between control and RNF126-depleted cells (Figure 6b). An equivalent increase of PDK protein-expression levels was observed in RNF126-depleted A549 cells (Supplementary Figure S3). Thus, RNF126 appeared to negatively regulate PDK1, PDK3 and PDK4 protein levels in both attached and detached cells.

Because PDKs phosphorylate three sites on PDH-E1α (Ser^232^, Ser^293^ and Ser^300^) [18, 19], the phosphorylation status of PDH-E1α was assessed using antibodies against PDH-E1α phosphorylated at specific sites (Figure 6c). RNF126 depletion enhanced phosphorylation at all three sites, which corresponded to increased PDK levels (Figure 6b and c). A PDK inhibitor, dichloroacetate (DCA), was tested to examine whether the effect of RNF126 depletion on ATP production (Figure 6d) was attributable to its effect on PDKs. DCA treatment of cells increased ATP production levels, and the effect was more apparent in detached cells than in attached cells (Figure 6d). Interestingly, DCA abrogated the effect of RNF126 depletion (Figure 6d), suggesting that RNF126 regulates cellular ATP levels by modulating PDK activities.

We then investigated the mechanism by which RNF126 regulates PDK protein levels. The mRNA levels of PDKs measured by real-time reverse transcription PCR increased in detached MDA-MB-231 cells (Supplementary Figure S4), resulting in increased PDK protein levels (Figure 6b). However, PDK mRNA levels were not strikingly different between control and RNF126-depleted cells in the attached and detached states (Supplementary Figure S4). Next, we examined the effect of a proteasome inhibitor, MG132, on the regulation of PDKs by RNF126. Treating the cells with MG132 increased PDK1, PDK3, and PDK4 protein levels in control cells (Figure 6e, shLuc/DMSO (dimethyl sulfoxide) versus shLuc/MG132), but not in RNF126-depleted cells in the attached state (Figure 6e, shRNF126/DMSO versus shRNF126/MG132). These results indicated that RNF126 promotes proteasomal degradation of the PDK proteins.

### RNF126 exhibited E3 ubiquitin ligase activity against PDKs

Protein ubiquitination is an important step in proteasomal degradation. E3 ubiquitin ligases recognize target proteins, and the RING domain of the ligases catalyzes the transfer of an ubiquitin moiety from an E2 ubiquitin ligase to the target proteins [20–22]. Because RNF126 contains a RING-like domain, we then analyzed whether RNF126 promotes the ubiquitination of PDKs.

**Figure 7:**
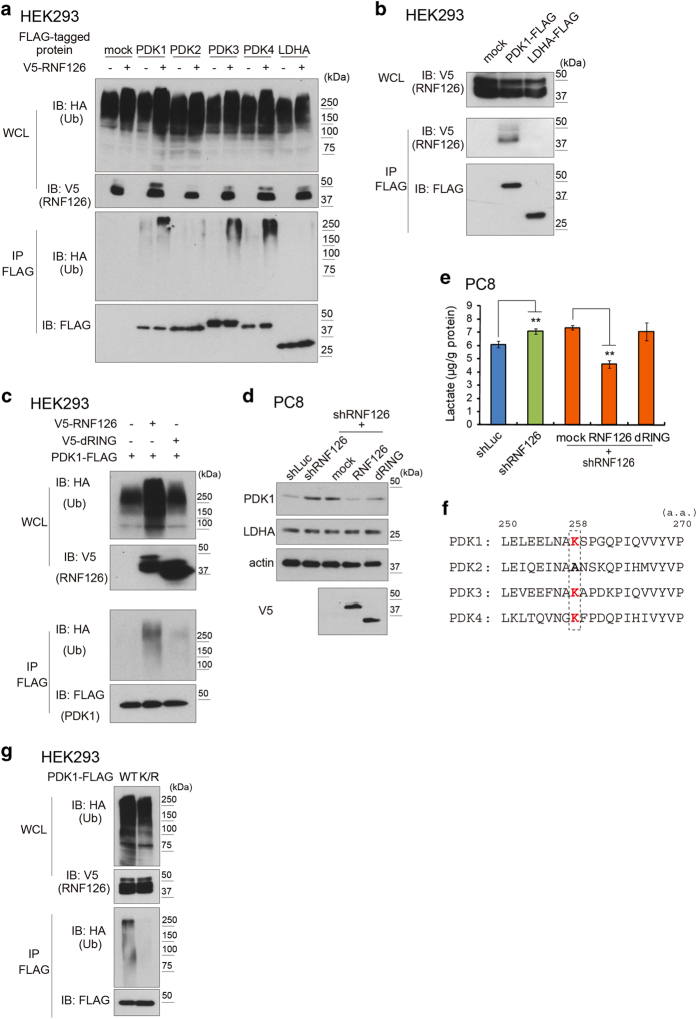
RNF126 is an E3 ubiquitin (Ub) ligase for PDKs. (**a**) Western blot analysis of the ubiquitination of PDK1-FLAG and LDHA-FLAG by V5-RNF126 in HEK293 cells. (**b**) Western blot analysis of FLAG immunoprecipitates (IPs) indicating that RNF126 interacts with PDK1, but not with LDHA, in HEK293 cells. (**c**) Western blot analysis of PDK1-FLAG ubiquitination demonstrating that the RING domain of RNF126 is necessary for PDK1 ubiquitination. (**d**) Forced expression of shRNF126-resistant RNF126 decreased PDK1 protein in attached PC8 cells that were depleted of endogenous RNF126. (**e**) Forced expression of shRNF126-resistant RNF126 decreased lactate production in attached cells depleted of endogenous RNF126. Error bars indicate the s.d. (*n*=3). The data were analyzed using a *t*-test. **P<0.01. (**f**) Amino-acid sequence alignment of the PDK family. (**g**) Western blot analysis showing ubiquitination of wild-type PDK1-FLAG or K/R mutant PDK1-FLAG. The data shown in (**a**–**e** and **g**) are representative of three independent experiments with similar results. HA, hemagglutinin; IB, immunoblotting; WCL, whole-cell lysate.

To examine this possibility, we expressed FLAG-tagged variants of PDKs or LDHA in human embryonic kidney 293 (HEK293) cells together with V5-tagged RNF126. Polyubiquitination of the target proteins was studied by western blot analysis. Indeed, polyubiquitination of the FLAG-tagged PDK1, PDK3 and PDK4, but not of PDK2 and LDHA, was observed specifically upon coexpression with V5-tagged RNF126 (Figure 7a). Furthermore, immunoprecipitation of V5-tagged RNF126 co-precipitated FLAG-tagged PDK1, but not FLAG-tagged LDHA (Figure 7b). FLAG-tagged PDK3 and PDK4 also bound to V5-tagged RNF126 (Supplementary Figure S5). Moreover, we confirmed that GST-tagged RNF126 recombinant protein is capable of catalyzing ubiquitination of a (His)_6_-tagged PDK1 recombinant protein *in vitro* (Supplementary Figure S6).

To confirm the necessity of the RING-like domain of RNF126 for PDK1 ubiquitination, we expressed a mutant RNF126 variant (dRING), in which the RING-like domain (amino acids 228–273) was deleted, in HEK293 cells and examined the effect of this mutant on the ubiquitination of FLAG-tagged PDK1 (Figure 7c). We found that the mutant was incapable of inducing ubiquitination of FLAG-tagged PDK1.

Next, we investigated the importance of the RING-like domain of RNF126 on the destabilization of endogenous PDK1 in human lung adenocarcinoma PC8 cells. We prepared RNF126-depleted PC8 cells and expressed either the V5-tagged RNF126 or V5-tagged dRING mutant proteins, whose mRNA sequences are resistant to the RNF126 shRNA. We observed decreased PDK1 protein expression in cells expressing V5-tagged RNF126, but not in cells expressing V5-tagged dRING, although no such effect was observed on LDHA (Figure 7d). The effect of the wild-type and mutant V5-tagged RNF126 proteins on PDK1 expression was reflected in the lactate production of the cells (Figure 7e).

Among the PDK family of proteins, PDK2 was barely ubiquitinated by RNF126, compared with the other PDKs (Figure 7a). Comparison of the amino-acid sequences of PDKs flanking the lysine residue that is a potential ubiquitination site revealed that the lysine residue at position 258 in PDK1 is conserved among all PDKs, except for PDK2 (Figure 7f). Substitution of this lysine in PDK1 by arginine (K258R) resulted in an inability of PDK1 to become ubiquitinated by RNF126 in HEK293 cells (Figure 7g).

### Elevated PDK levels accounted for the effect of RNF126 on tumor growth in mice

RNF126 negatively regulated PDK protein levels and was necessary for the tumorigenicity of cancer cells in mice (Figure 2a and b). However, RNF126 may target other proteins responsible for the effect of RNF126 on tumor growth in mice. Therefore, we investigated whether the increased levels of PDKs in MDA-MB-231 cells contribute to the reduced tumorigenicity observed with RNF126-depleted cells, as shown in Figure 2a. We chose PDK1 as a representative of the PDK family of proteins because only PDK1 can phosphorylate all three sites in PDH-E1α, whereas other PDKs phosphorylate only Ser^293^ and Ser^300^ of PDH-E1α [18, 19].

**Figure 8:**
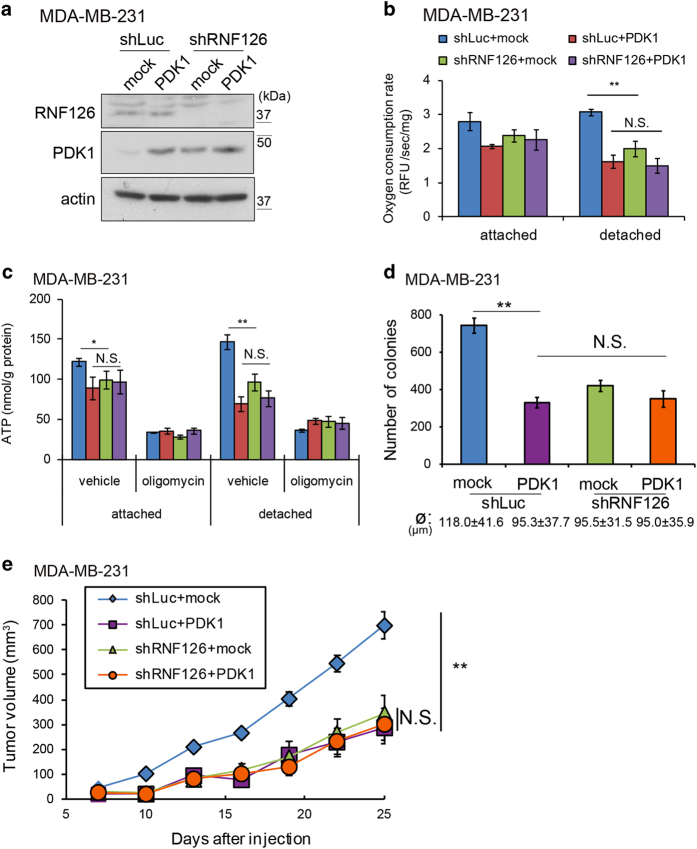
RNF126 promotes tumorigenicity in a PDK-dependent manner. (**a**) Western blot analysis of exogenous PDK1 expression in control and RNF126-depleted MDA-MB-231 cells. (**b** and **c**) Control and RNF126-depleted MDA-MB-231 cells expressing exogenous PDK1 were cultured under attached or detached conditions, after which they were assayed to determine their oxygen consumption rates (**b**) and cellular ATP contents (**c**). (**d**) The number of colonies formed in soft agar by control and RNF126-depleted MDA-MD-231 cells expressing exogenous PDK1. The diameter (ø) of the counted colonies is indicated as the average±s.d. (**e**) Tumor growth following subcutaneous implantation of control and RNF126-depleted MDA-MB-231 cells expressing exogenous PDK1 in mice. In (**b–d**), the error bars indicate the s.d. (*n*=3). The data were analyzed using a *t*-test. **P<0.01. NS, not significant. The data shown are representative of three independent experiments with similar results. In (**e**), the error bars indicate the s.e.m.; *n*=8 from two independent experiments (*n*=4 and *n*=4, respectively); the data shown were analyzed using the Mann–Whitney *U*-test. **P<0.01. RFU, relative fluorescence units.

PDK1 was expressed in control and RNF126-depleted MDA-MB-231 cells (Figure 8a). Under the detached condition, exogenous PDK1 expression decreased oxygen consumption and ATP levels in MDA-MB-231 cells to those observed in RNF126-depleted cells. However, further RNF126 depletion did not decrease oxygen consumption or ATP levels in cells expressing exogenous PDK1 (Figure 8b and c). Then, we investigated the colony-forming ability and tumorigenicity of cells expressing exogenous PDK1. Exogenous PDK1 expression decreased the colony-forming capacity of control cells in soft agar to the levels of RNF126-depleted cells (Figure 8d). Similarly, cells expressing exogenous PDK1 developed into tumors with ~70% smaller volumes than observed with the corresponding control cells at day 25 (Figure 8e), and PDK1 overexpression in RNF126-depleted cells did not decrease the tumor volumes further (Figure 8e). These results strongly suggested that the effect of RNF126 on colony formation in soft agar and tumor growth reflects its effects on PDK proteins.

### ERK-mediated RNF126 and PDK regulation in normal and cancer cells

**Figure 9:**
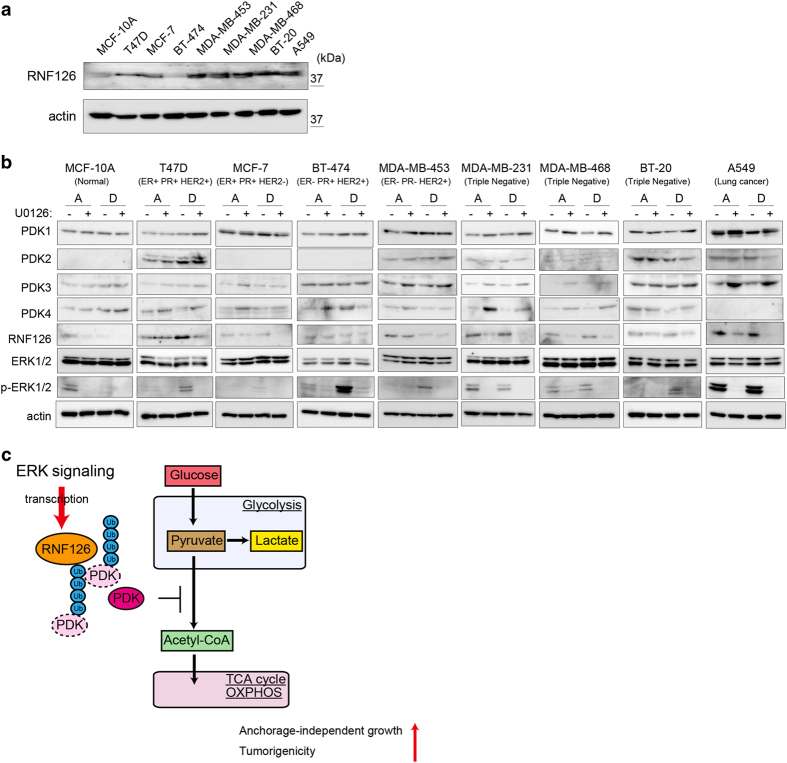
ERK-dependent expression of PDKs and RNF126 in normal epithelial and breast cancer cells, in the attached or detached state. (**a**) Western blot analysis of RNF126 and actin in cells in the attached state. (**b**) Western blot analysis of the phosphorylation of PDKs, RNF126 and ERK in cells cultured under attached or detached condition, in the presence or absence of U0126 (10 μm). The data shown in (**a** and **b**) are representative of three independent experiments with similar results. (**c**) Schematic illustration showing that RNF126 serves as a regulator of the metabolic shift between glycolysis and the TCA cycle by degrading PDK. ER, estrogen receptor; p, phosphorylated; PR, progesterone receptor.

Finally, we explored ERK-mediated RNF126 and PDK expression in attached and detached cells using normal mammary epithelial MCF-10A cells, seven breast cancer cell lines including MDA-MB-231 cells and lung cancer A549 cells. All of the cancer cell lines (except for BT-474 cells) expressed RNF126 at higher levels than did MCF-10A cells in the attached state (Figure 9a). In normal epithelial MCF-10A cells, U0126 treatment decreased phosphorylated ERK and RNF126 levels, even in the attached condition. The expression of PDK4 (but not that of other PDKs) increased following U0126 treatment in the attached condition. Thus, ERK signaling promotes RNF126 expression even in attached normal epithelial MCF-10A cells, although ERK-mediated PDK regulation is limited in these cells.

When MCF-10A cells were grown in the detached condition, phosphorylated ERK and RNF126 levels dropped strikingly (Figure 9b). In contrast, most cancer cells showed maintained or increased phosphorylated ERK and RNF126 levels under the detached condition. U0126 treatment decreased RNF126 levels in detached cancer cells, except in BT-474 and MDA-MB-453 cells, where U0126 treatment did not affect RNF126 levels. Among the breast cancer cell lines studied, ERK-mediated PDK regulation was more apparent in triple-negative breast cancer cell lines (MDA-MB-231, MDA-MB-468 and BT-20) than in other types of breast cancer cell lines. Thus, the impact of ERK-mediated PDK regulation depends on the cell type, even in cancer cells. Taken together, these data indicated that ERK phosphorylation and RNF126 are lost under the detached condition in normal cells, but are maintained in most cancer cells. Because RNF126 depletion attenuates tumorigenicity and colony-forming ability in soft agar, the maintained expression of RNF126 in the detached state appears to be important for tumorigenicity in cancer cells.

## Discussion

In this study, RNF126 was characterized as a new factor regulating the balance between glycolysis and OXPHOS, as summarized in Figure 9c. RNF126 acts as an E3 ubiquitin ligase of PDKs and leads to their proteasomal degradation. Because PDKs phosphorylate and inhibit PDH activity, which mediates the conversion of pyruvate to acetyl-CoA, the downregulation of PDKs by RNF126 promotes the flux of metabolites from glycolysis into the TCA cycle. PDK1, PDK3 and PDK4, but not PDK2, were ubiquitinated by RNF126. The lysine residue at position 258 (K^258^) was conserved among most ubiquitinated PDKs, but not in PDK2. RNF126 depletion in cells increased PDK1, PDK3 and PDK4 protein levels, increased lactate production without changing the total glucose consumption and decreased ATP production by OXPHOS. In our previous study, *RNF126* attracted our attention as a gene that promotes cell growth during normoxia, but not during hypoxia, by a genetic screen of PC8 cells using an shRNA library [13]. The shRNA sequences for *RNF126* accumulated to a greater extent in cells grown under hypoxic culture conditions than those grown under normoxic conditions. This result can be explained if PDK1 is the target of RNF126, reducing cell growth, because the effect of RNF126 on PDK1 can become diminished with enhanced PDK1 expression following hypoxia induced by the hypoxia-inducible factor-1 transcription factor [16, 17].

RNF126 may also regulate cell growth by targeting proteins other than PDKs. Recently, RNF126 has been reported to be involved in diverse biological phenomena, such as the cell cycle, membrane trafficking, B-cell maturation and protein quality control [23–27]. Although it was difficult to completely exclude the involvement of other target proteins in our assays, PDKs appeared to be the major targets of RNF126 involved in cell growth regulation during colony formation in soft agar and tumor formation in mice. For example, an increase in PDK1 levels by exogenous PDK1 expression in the cells suppressed colony formation and tumor growth, similar to the effects of RNF126 depletion (Figure 8d and e). Enhanced expression of PDK1 in RNF126-depleted cells did not further suppress tumor growth. Furthermore, RNF126 depletion did not affect mitochondrial function and tumorigenicity in PDK1-depleted MDA-MB-231 cells (Supplementary Figure S7). Thus, our results supported the concept that RNF126 regulates cell growth by targeting PDKs.

Because more apoptotic cells were observed in tumors formed by RNF126-depleted cells than those formed by control cells, sufficient activity levels of the TCA cycle and OXPHOS are presumably important for anoikis resistance and cell survival. Previously, RNF126 was reported to regulate the cell cycle by inducing proteasomal degradation of the cyclin inhibitor p21/Cip1 [25]. However, RNF126 depletion did not affect p21/Cip1 protein levels in the MDA-MB-231 and A549 cells used under our culture conditions (Supplementary Figure S8). Thus, p21/Cip1 was not a major target of RNF126 in terms of regulating cell growth in our system, although RNF126-dependent p21/Cip1 regulation may operate in a cell context-dependent manner.

Despite the modest effect of RNF126-KD on cell growth in monolayers, marked suppression of colony formation in soft agar was observed, and, accordingly, tumor formation in mice was also reduced. The colony-forming ability of tumor cells in soft agar requires the acquisition of anoikis resistance, which is also required for tumor formation and metastatic colony formation. Oncogenic signals contribute to anoikis resistance, and the ERK signaling pathway is especially important for the acquisition of anoikis resistance [2, 28]. The ERK pathway enhanced RNF126 expression, and RNF126 depletion impaired anoikis resistance, without affecting the ERK signaling pathway itself. Therefore, RNF126 may be a key factor in ERK signaling-mediated anoikis resistance in cancer cells. In BT-474 and MDA-MB-453 cells, U0126 treatment decreased the phosphorylation levels of ERK1/2, but did not affect RNF126 expression (Figure 9b). Other signaling pathway(s) might regulate RNF126 expression in these cells.

Reprograming of metabolic activity occurs when cells detach from the ECM [10, 11, 29]. For example, PDK4 expression is induced in immortalized, non-tumorigenic mammary epithelial cells (MCF-10A) upon detachment from the ECM, which inhibits metabolic flux from glycolysis to the TCA cycle, coupled with OXPHOS, and limits the energy supply provided by OXPHOS [10, 11]. Activation of the ERK/MAPK pathway inhibits cellular PDK4 expression and contributes to cell survival following detachment from the ECM [10]. In our experiments, different patterns of PDK expression were observed in individual cell lines under the detached condition (Figure 9b). Thus, the expression of PDKs in response to the detached condition may depend on the cell type and the intracellular signals activated in the cells. In MDA-MB-231 cells, the PDK4 protein level was maintained, whereas PDK4 mRNA levels increased strikingly in detached MDA-MB-231 cells (Figure 6b and Supplementary Figure S4). Thus, PDK levels appear to be intricately regulated in transcriptional, translational and post-translational manners in detached cells. Moreover, the activities of PDKs have crucial roles in regulating the flux between glycolysis and the TCA cycle. The survival of detached cells depends on the TCA cycle and OXPHOS more so than do attached cells; we propose that this is the main reason that growth and survival of non-adherent cells are more sensitive to RNF126 depletion than are adhered cells. Increased glucose consumption during normoxia is a hallmark of aggressive cancer cells; this phenomenon is known as the Warburg effect [30]. Even though RNF126 regulates influx from glycolysis to the TCA cycle, it did not affect the net glucose consumption of the cells. Cellular metabolism proceeds through complex interconnected systems to maintain homeostasis. In this study, we focused on the roles of RNF126 in glycolysis and the TCA cycle. Indeed, RNF126 and PDKs regulate the metabolic flux to mitochondria, and other metabolic pathways can affect net metabolite production and ATP levels. For example, autophagy supplies amino acids when cells are starved, and pyruvate can be produced from alanine-by-alanine transaminase [31]. This might compensate for reduced glycolysis and help to maintain lactate production and mitochondrial metabolism in detached MDA-MB-231 cells (Figure 5). Even though these feedback systems compensate for metabolic alterations in detached cells, PDK levels regulated by RNF126 appear to have a critical role in cellular metabolism.

Results from many studies have shown that the depletion of PDK1 alone significantly hampers tumorigenicity [16, 17, 32, 33] and we confirmed this finding in MDA-MB-231 and A549 cells (Supplementary Figure S9). Thus, among the PDK family members, PDK1 has an indispensable role in tumorigenicity. However, interestingly, PDK1 overexpression also affects tumorigenicity in MDA-MB-231 cells (Figure 8e). In addition, overexpression of RNF126, but not its RING domain deletion mutant (dRING), caused decreased protein expression of PDK1, PDK3 and PDK4; colony formation in soft agar; tumorigenicity; and phenocopying of PDK1 KD (Supplementary Figure S10). RNF126 is indeed necessary for tumorigenicity, but excess expression of RNF126 affects tumorigenicity and PDK1 expression, at least in MDA-MB-231 cells. This biphasic inhibitory effect of PDK1 and RNF126 may indicate that growth and survival of tumor cells are highly sensitive to fine-tuning of the PDK1-mediated metabolic pathway. Indeed, the regulation of PDK1 activity is well-orchestrated in cancer cells: mRNA levels of *PDK1* are regulated by hypoxia-inducible factor-1 and Myc [16, 17, 34], whereas the phosphorylation of PDK1 by oncogenic tyrosine kinases promotes PDK1 activity [32]. In addition, RNF126 ensures that PDK1 protein levels are maintained by proteasomal degradation. Thus, RNF126 depletion may diminish tumorigenicity of cells mainly by affecting the fine-tuning of PDK1 activity in cancer cells.

Many studies have focused on the effects of enhanced glycolysis in cancer cells, such as the Warburg effect. However, recently, mitochondrial function and oxidative phosphorylation have been implicated in cancer proliferation, metastasis and resistance to driver-oncogene ablation [35–37]. Thus, the role of aerobic metabolism in tumor malignancy has attracted increasing attention. In this study, we revealed the molecular mechanism of RNF126 on anchorage-independent cancer growth and OXPHOS regulation. Most breast cancer cell lines expressed RNF126 at higher levels than did mammary epithelial MCF-10 A cells (Figure 9a). We performed database analyses on RNF126 expression in clinical samples, and several data sets showed that RNF126 expression increased in breast cancer tissues (compared with that measured in normal tissues), and higher RNF126 expression was associated with decreased distant metastasis-free survival in patients with breast cancer (Supplementary Figure S11). Although excess expression of RNF126 decreased the tumorigenicity in MDA-MB-231 cells (Supplementary Figure S10), RNF126 expression at appropriate levels in the tumor microenvironment might worsen the prognosis of cancer patients. Future studies will uncover the role of RNF126-mediated OXPHOS regulation in tumorigenesis and clinical problems, such as the acquisition of refractoriness to therapy.

In summary, we identified RNF126 as a new regulator of the PDK family of proteins that control the influx of pyruvate into the TCA cycle. This may be an Achilles’s heel for cancer cells that have acquired anoikis resistance.

## Materials and Methods

### Cell culture

The human embryonic kidney cell line HEK293, the mammary epithelial cell line MCF-10A, the breast cancer cell line MDA-MB-231, T47D, MCF-7, BT-474, MDA-MB-453, MDA-MB-468, BT-20, and the lung adenocarcinoma cell line A549 were purchased from the American Type Culture Collection (Manassas, VA, USA). Human lung adenocarcinoma PC8 cells [38] were kindly gifted from the National Cancer Center (Tokyo, Japan). MCF-10A cells were cultured in Dulbecco’s modified Eagle's medium/F12 medium containing 1% fetal bovine serum, non-essential amino-acid solution (Life Technologies, Carlsbad, CA, USA), 20 ng ml^−1^ human EGF (Peprotech, Rocky Hill, NJ, USA), 10 μg ml^−1^ bovine pituitary extract (Thermo Fisher Scientific Inc., Waltham, MA, USA), 500 μg ml^−1^ hydrocortisone (Nalcalai Tesque, Kyoto, Japan), 10 μg ml^−1^ human insulin solution (Sigma-Aldrich, St Louis, MO, USA), 100 U ml^−1^ penicillin and 100 μg ml^−1^ streptomycin (Sigma-Aldrich). Other cells were cultured in Dulbecco’s modified Eagle's medium (MDA-MB-231, A549, MCF-7), Dulbecco’s modified Eagle's medium high-glucose (HEK293, T47D, MDA-MB-453, MDA-MB-468) or RPMI (PC8, BT-474, BT-20) containing 10% fetal bovine serum, 100 U ml^−1^ penicillin and 100 μg ml^−1^ streptomycin at 37 °C in humidified incubator with 5% CO_2_. For suspension cultures, cells were seeded on Ultra-Low Cluster Plates (Corning Inc., Corning, NY, USA). For experiments on the ERK signaling pathway, cells were treated with the MEK inhibitor U0126 (10 μm; Wako, Tokyo, Japan) for 24 h.

### Vector construction

The shRNA sequences used in this study were as follows: shLuc, 5′-GCAUCACGUACGCGGAAUACCGAAGUAUUCCGCGUACGUGAUG -3′ (*RNF126* no. 1) and 5′-GCUUUGAAAUAAACGGACGUUCGAAAACGUCCGUUUAUUUCAAAGC -3′ (*RNF126* no. 2). The DNA versions of the targeted gene sequences were subcloned into the pENTR/U6 TOPO Vector (Life Technologies, Carlsbad, CA, USA) before being transferred via recombination into the lentivirus vector, pLenti6 BLOCK iT (Life Technologies). Human *PDK1-4*, *RNF126* and *LDHA* cDNAs were amplified from MDA-MB-231 cells by reverse transcription PCR. Constructs expressing mutant RNF126 or PDK1 were prepared using a PCR-based method. These cDNAs were subcloned into pENTR/D-TOPO (Life Technologies) before being transferred into the lentivirus vector pLenti6, or the mammalian expression vector, pcDNA3.2 via recombination, as described previously [13, 39, 40]. The lentiviral vectors were generated and used according to the manufacturer’s instructions. To generate reporter plasmids containing the RNF126 promoter, human RNF126 promoter regions were amplified by PCR using MDA-MB-231 genomic DNA as a template and cloned into the pGL3 Basic Vector (Promega, Madison, WI, USA). The nucleotide positions in the RNF126 promoter were numbered based on the first nucleotide of the open reading frame of RNF126 mRNA. Mutations of one or both of the potential ELK1 binding sites between −1 880 and −1 860 of the RNF126 promoter were introduced into the reporter vector by a PCR-based method, which involved the replacement of 5′- GA-3′ with 5′- AG-3′ in the ELK1 binding site.

### Cell growth assay

Cells (1×10^4^) were seeded into a plastic tissue culture dish and cultured at 37 °C in a humidified CO_2_ incubator for 7 days. The cells were counted periodically using a Coulter Counter (Beckman Coulter, Fullerton, CA, USA).

### Soft agar assay

Cells (2×10^4^ for MDA-MB-231; 1×10^4^ for A549) were cultured in media containing 0.36% Bacto agar (Difco, Franklin Lakes, NJ, USA) for 21 days and colonies with a diameter ⩾50 μm (MDA-MB-231) or ⩾100 μm (A549) were counted.

### Western blot analysis

Cells were lysed with lysis buffer and centrifuged at 20 000 *g* for 15 min at 4 °C. The supernatants were collected and the total protein contents were measured using a Bradford assay (Bio-Rad, Hercules, CA, USA). Lysates were separated by sodium dodecyl sulfate-polyacrylamide gel electrophoresis, transferred to membrane filters and analyzed by western blotting using an anti-V5 mouse antibody (Life Technologies), an anti-HA mouse antibody (Life Technologies), an anti-FLAG epitope M2 antibody (Sigma-Aldrich), an anti-actin mouse antibody (Chemicon, Billerica, MA, USA), an anti-Erk1/2 rabbit antibody (Cell Signaling Technology, Danvers, MA, USA), an anti-phospho-Erk1/2 (Thr202/Tyr204) rabbit antibody (Cell Signaling Technology), an anti-PDK1 rabbit antibody (Lifespan Biosciences, Seattle, WA, USA), an anti-PDK2 rabbit antibody (GeneTex, Irvine, CA, USA), an anti-PDK3 rabbit antibody (GeneTex), an anti-PDK4 rabbit antibody (GeneTex), an anti-LDHA rabbit antibody (Novus Biologicals, Littleton, CO, USA), an anti-PDH-E1α mouse antibody (Life Technologies), anti-PDH-E1α (pSer^232^, pSer^293^, pSer^300^) rabbit antibodies (Calbiochem, San Diego, CA, USA), an anti-poly(ADP-ribose) polymerase rabbit antibody (Cell Signaling Technology), an anti-caspase-3 rabbit antibody (Cell Signaling Technology), an anticleaved caspase-3 rabbit antibody (Cell Signaling Technology) or an anti-RNF126 rat antibody. The anti-RNF126 rat monoclonal antibody was generated by Eurofins Genomics (Tokyo, Japan) using a (His)_6_-RNF126 N-terminus (amino acids 1–214) fusion as an immunogen, and the supernatant of the hybridoma was used. Densitometry of obtained signals was performed using the ImageJ software (National Institutes of Health, Bethesda, MD, USA).

### Immunoprecipitation

HEK293 cells were co-transfected with expression plasmids encoding an N-terminally V5-tagged RNF126 or its dRING mutant, and C-terminally FLAG-tagged PDKs 1, 2, 3 or 4, or lactate dehydrogenase A. To detect ubiquitination, hemagglutinin-tagged ubiquitin was also expressed in HEK293 cells. Constructs were transfected into cells using Lipofectamine 2000 (Life Technologies). At 24 h post-transfection, the cells were lysed with lysis buffer (1% NP-40, 50 mm Tris (pH 8.0), 150 mm NaCl) and centrifuged at 20 000 *g* for 15 min at 4 °C. The supernatants were collected and incubated with anti-FLAG M2 antibody-conjugated beads (Sigma-Aldrich). Beads were washed and bound proteins were eluted and detected by western blot analysis.

### RNA isolation, reverse transcription and real-time PCR

Total RNA was isolated from cells using TRIzol (Life Technologies) and subjected to reverse transcription (RT) using Superscript II (Life Technologies) and random primers. The RT products were then analyzed by real-time PCR in a 7500 Real-Time PCR System (Applied Biosystems (ABI), Foster City, CA, USA) using SYBR Green PCR Master Mix (ABI) and the following specific primers: ACTB (β-actin) sense, 5′-GAGCTTCCGGAAGAGACCAG -3′ and antisense, 5′- GGATCTCGAAGCTGTCATCG-3′; RNF126 sense, 5′- GAGCTTCCGGAAGAGACCAG-3′ and antisense, 5′- GGATCTCGAAGCTGTCATCG-3′; PDK1 sense, 5′-TCCTGTCACCAGCCAGAATG -3′ and antisense, 5′-CTTCCTTTGCCTTTTCCACC -3′; PDK2 sense, 5′- TCCTGTCACCAGCCAGAATG-3′ and antisense, 5′-CTTCCTTTGCCTTTTCCACC -3′; PDK3 sense, 5′-TCCTGTCACCAGCCAGAATG -3′ and antisense, 5′-CTTCCTTTGCCTTTTCCACC -3′; PDK4 sense, 5′-TCCTGTCACCAGCCAGAATG-3′ and antisense, 5′-CTTCCTTTGCCTTTTCCACC -3′. The PCR products were sequenced and their homogeneity was confirmed by dissociation temperature monitoring of SYBR Green I fluorescence.

### Reporter assay

The reporter plasmid used contains the firefly luciferase gene under the control of the RNF126 promoter. A pRL vector expressing *Renilla* luciferase (Promega) served as an internal control. MDA-MB-231 cells (5×10^4^ per well) were seeded into 24-well plates and co-transfected with a reporter plasmid (100 ng) and the internal control vector (10 ng) in the presence or absence of U0126 (10 μm). Transfection was performed using Lipofectamine 2000 (Life Technologies). Luciferase activity was measured using the Dual Luciferase Reporter Assay System (Promega) according to the manufacturer’s instructions. Luminescence was measured in a GloMax 20/20 luminometer (Promega).

### ChIP assay

ChIP assays were performed using the SimpleChIP Plus Enzymatic Chromatin IP Kit (Cell Signaling Technology) per the manufacturer’s instructions. A rabbit anti-ELK1 antibody (Santa Cruz Biotechnology, Dallas, TX, USA) and a control IgG (Cell Signaling Technology) were used for immunoprecipitation. Precipitated DNAs were subjected to real-time PCR using the following specific primers: RNF126 promoter region from −1 903 to −1 836: sense, 5′-CTGGATTCGCGCTGCTGCAAAG -3′ and antisense, 5′-AATGTCCAGAGGTTGGGGACAG-3′; RNF126 promoter region from −304 to −212 region: sense, 5′-CGCGCACGCCCCGCTTCTCGTCTC -3′ and antisense, 5′-GGCCGTTGGGCGGGGCTCCAAGCG-3′.

### Metabolomic analysis

Capillary electrophoresis-time of flight/mass spectrometry analysis of glycolytic and TCA cycle metabolites in cells was performed by Human Metabolome Technologies Inc. (Tsuruoka, Japan).

### Measurement of lactic acid

Cells were seeded in 24-well plates (1×10^5^/well) in triplicate. Conditioned medium was collected after 6 h and the lactic acid contents thereof were measured using an l-Lactic Acid Kit (R-Biopharm, Darmstadt, Germany). The values obtained were normalized to protein concentrations that were determined using a Bradford Assay Kit (Bio-Rad), as described previously [41].

### Measurement of ATP

Cells were cultured in the presence or absence of the OXPHOS inhibitor, oligomycin (5 μg ml^−1^; Sigma-Aldrich), or the PDK inhibitor, DCA (500 μm; Wako) for 2 h, and ATP levels were determined using the ATP Bioluminescence Assay Kit CLS II (Roche Applied Science, Penzberg, Germany). ATP levels were normalized to total protein concentrations, which were determined using a Bradford Assay Kit (Bio-Rad), as previously described [41].

### Measurement of glucose consumption

Cells were seeded in 6-well plates (4×10^5^ per well) in triplicate. Conditioned medium was collected after 24 h, and the glucose concentration therein was measured using a Glucose Assay Kit (Sigma-Aldrich). Glucose consumption levels were normalized to total protein concentrations, which were determined using a Bradford Assay Kit (Bio-Rad).

### Measurement of oxygen consumption

Cells were seeded in 96-well plates (2×10^4^ per well) in triplicate. Fluorescein accumulation, which indicates oxygen consumption, was measured every 90 s using the MitoXpress-Xtra-HS Reagents (Luxcel Biosciences, Cork, Ireland) according to the manufacturer’s instructions, with a FLUOstar OPTIMA luminometer (BMG Labtech, Ortenberg, Germany). Fluorescein accumulation rates from 0 to 3 600 s were normalized to total protein concentrations, which were determined using a Bradford Assay Kit (Bio-Rad).

### Tumor growth assay

Tumor growth experiments were conducted according to the institutional ethical guidelines for animal experiments and the safety guidelines for gene manipulation experiments (The Institute of Medical Science, University of Tokyo, Tokyo, Japan). The tumorigenicity of cells was examined using 6-week-old female BALB/c nude mice (CLEA Japan, Tokyo, Japan). Briefly, 1×10^6^ (MDA-MB-231) or 5×10^5^ (A549) cells were injected subcutaneously into the dorsal side of the mice. Subsequently, the implanted tumors were blindly measured with a caliper on the indicated days and their volumes were calculated using the formula: *V*=(*L*x*W*^2^)/2, where *V* is the volume (mm^3^), *L* is the biggest tumor diameter (mm) and *W* is the smallest tumor diameter (mm).

### Immunostaining

Animals were killed 25 days after inoculation, and their tumors were embedded in the OCT compound (Sakura Finetek, Torrance, CA, USA) and stored at −80 °C. Frozen sections (10 μm thick) were fixed in 4% paraformaldehyde/phosphate-buffered saline and subjected to immunostaining. Immunostaining was performed using a rabbit anticleaved caspase-3 antibody (Cell Signaling Technology), as described previously [39]. The nuclei were counterstained with Hoechst 33342, and the sections were observed by confocal microscopy (Carl Zeiss, Oberkochen, Germany).

### Statistical analysis

We compared two groups using a two-sided *t*-test or the Mann–Whitney *U*-test.
