# Supplementary material for: The ERK signaling target RNF126 regulates anoikis resistance in cancer cells by changing the mitochondrial metabolic flux
Source: Cell Discov. 2016 Jul 26;2:16019–. doi: 10.1038/celldisc.2016.19 (PMC4960523; doi:10.1038/celldisc.2016.19)
Supplement: Supplementary Information [file celldisc201619-s1.pdf]

Figure S1

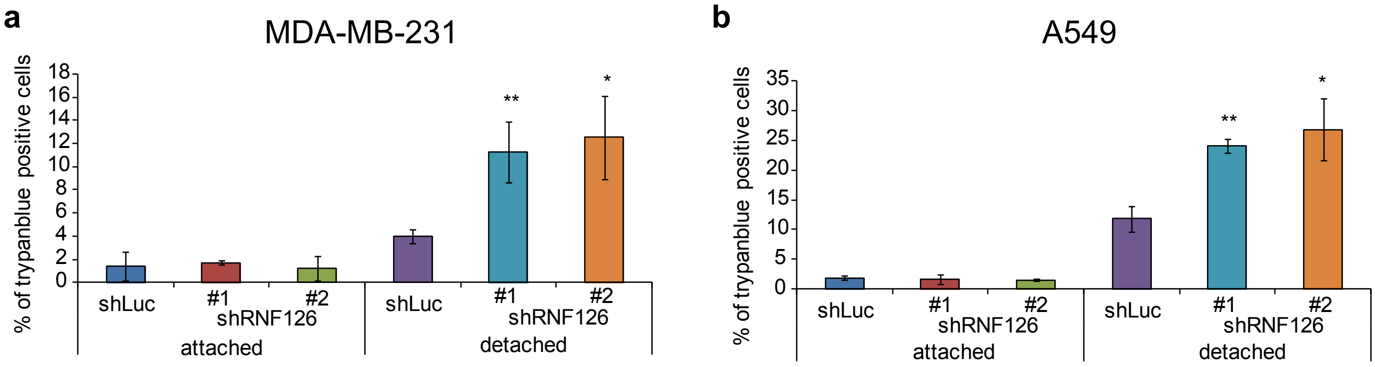

Figure S1 RNF126 regulates anoikis.
(a, b) Trypan blue-exclusion assay of control and RNF126-depleted MDA-MB-231 (**a**) and A549 cells (**b**) that had been cultured under attached or detached conditions for 96 h. The data shown are from 3 independent experiments. Error bars indicate the standard deviations.

Figure S2

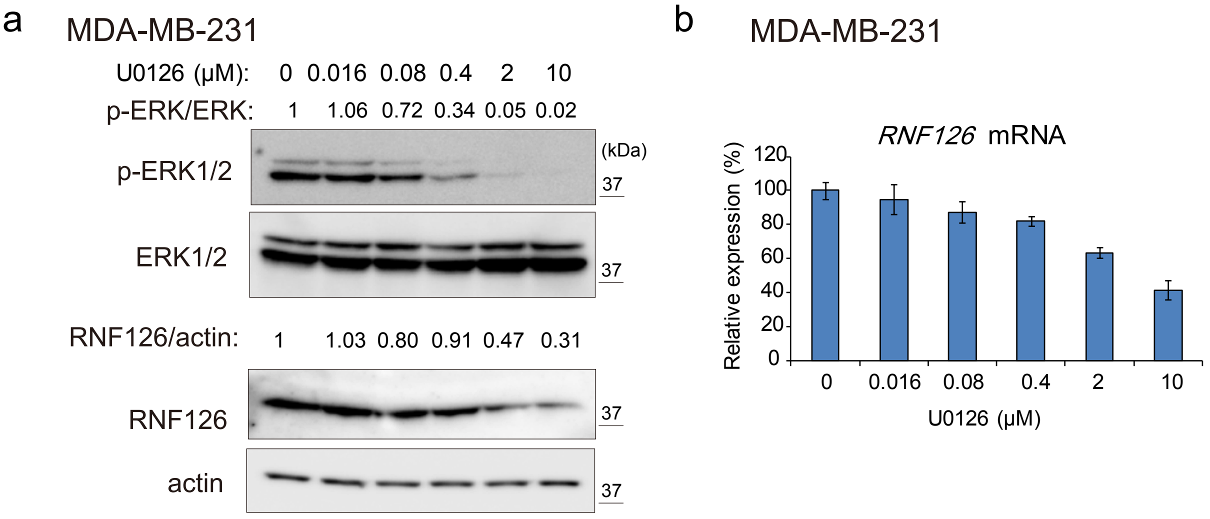

Figure S2 ERK-dependent RNF126 expression in MDA-MB-231 cells.
(**a**) Western blot analysis of RNF126, ERK1/2, phosphorylated-ERK1/2, and actin in attached MDA-MB-231 cells treated with U0126 at the indicated concentration for 24 h. Results of the densitometric analysis of bands in western blots are presented.
(**b**) RT-PCR analysis of RNF126 mRNA in U0126-treated MDA-MB-231 cells. The error bars indicate the standard deviations (n = 3).
The data shown in **a** and **b** are representative of 3 independent experiments with similar results.

Figure S3

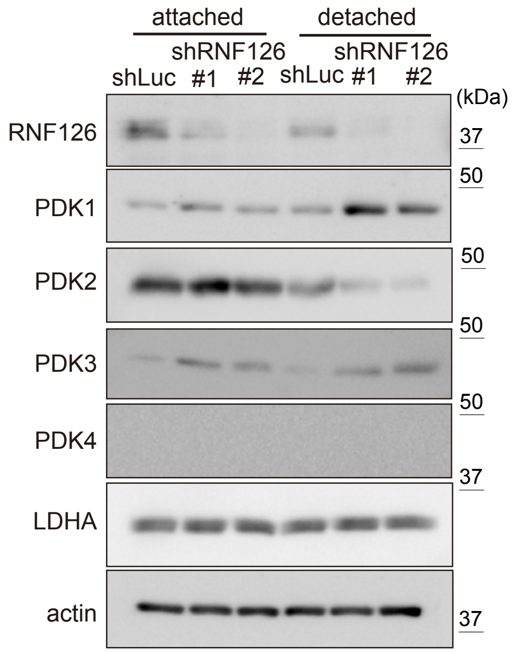

Figure S3 RNF126 regulates PDK protein levels in A549 cells.
Western blot analysis of PDKs and LDHA expression in control and RNF126-depleted A549 cells cultured under attached or detached conditions. PDK4 was undetectable in A549 cells. The data shown are representative of 3 independent experiments with similar results.

Figure S4

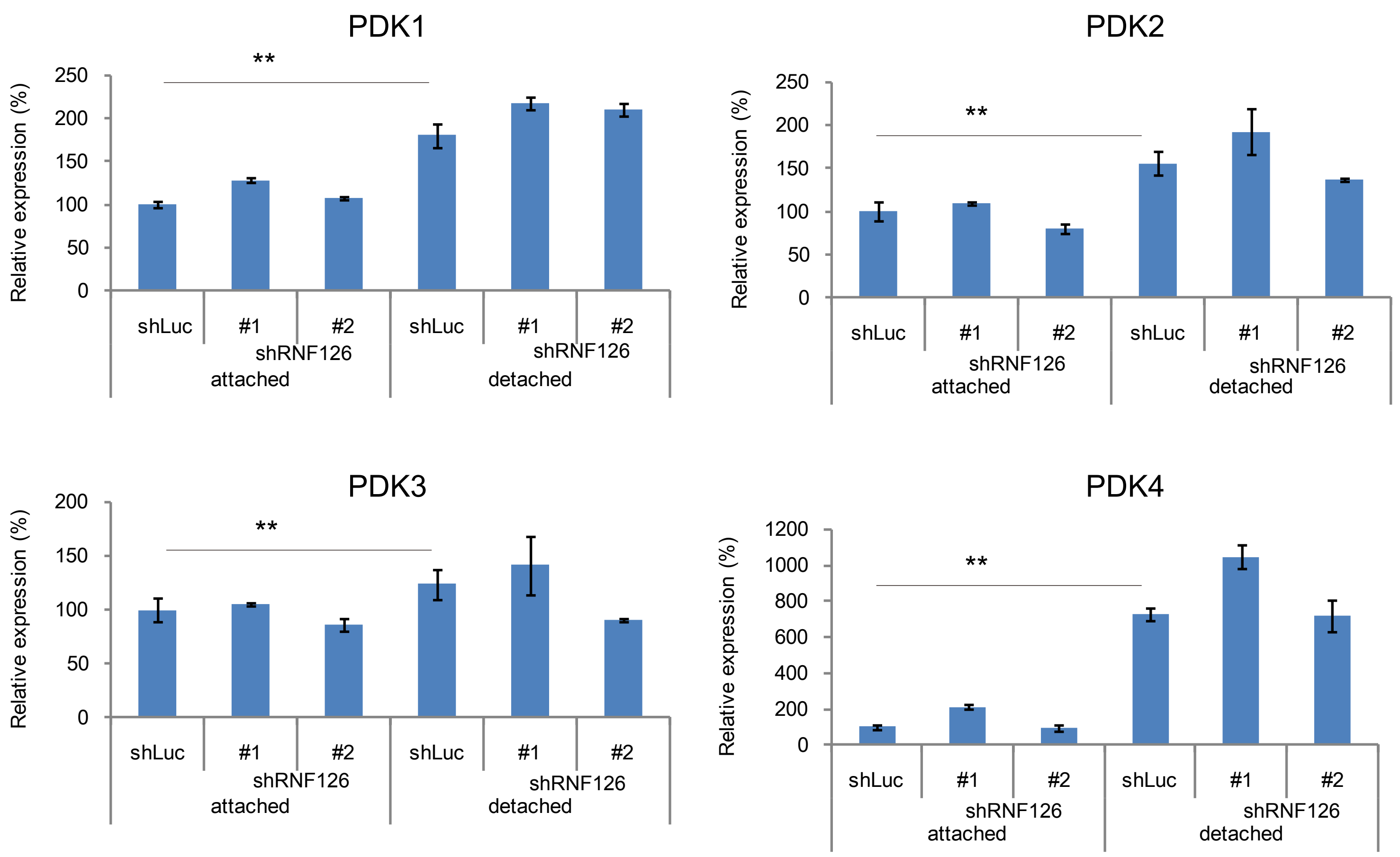

Figure S4 RT-PCR analysis of PDK mRNA in control and RNF126-depleted cells.
Error bars indicate standard deviations (n = 3); data were analyzed using the t-test. **p < 0.01. The data shown are representative of 3 independent experiments with similar results.

Figure S5

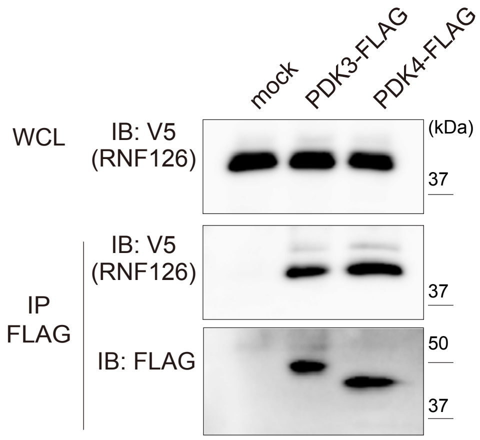

Figure S5 RNF126 interacts with PDK3 and PDK4.
Western blot analysis of FLAG immunoprecipitates, indicating that RNF126 interacts with PDK3 and PDK4 in HEK293 cells.

Figure S6

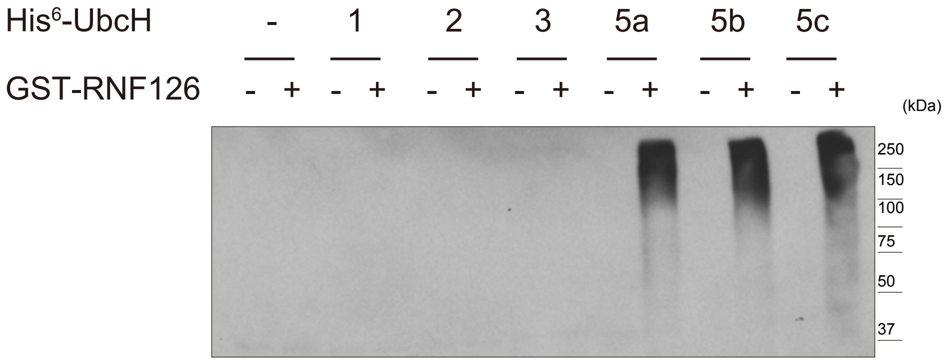

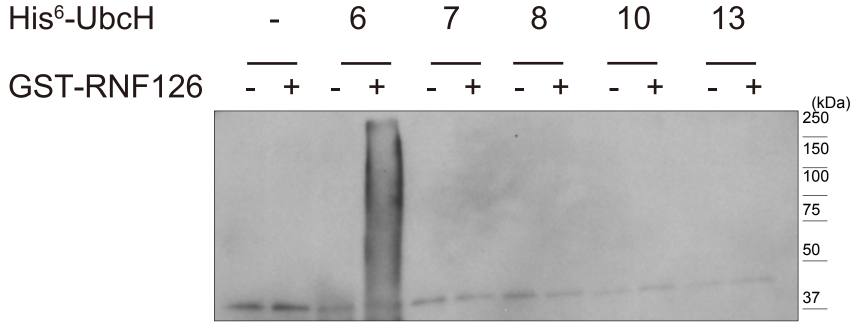

Figure S6 In vitro ubiquitination of PDK1 by RNF126.
RNF126 ubiquitinates PDK1 in vitro. (His)6-PDK1 was incubated with the indicated E2 proteins, biotin-ubiquitin, and other components for ubiquitination, in the presence of GST or GST-RNF126. Ubiquitinated proteins were detected using streptavidin-HRP. Note that RNF126 uses UbcH5a, 5b, 5c, and 6 as the E2 protein in vitro. The data shown are representative of 3 independent experiments with similar results.

Figure S7

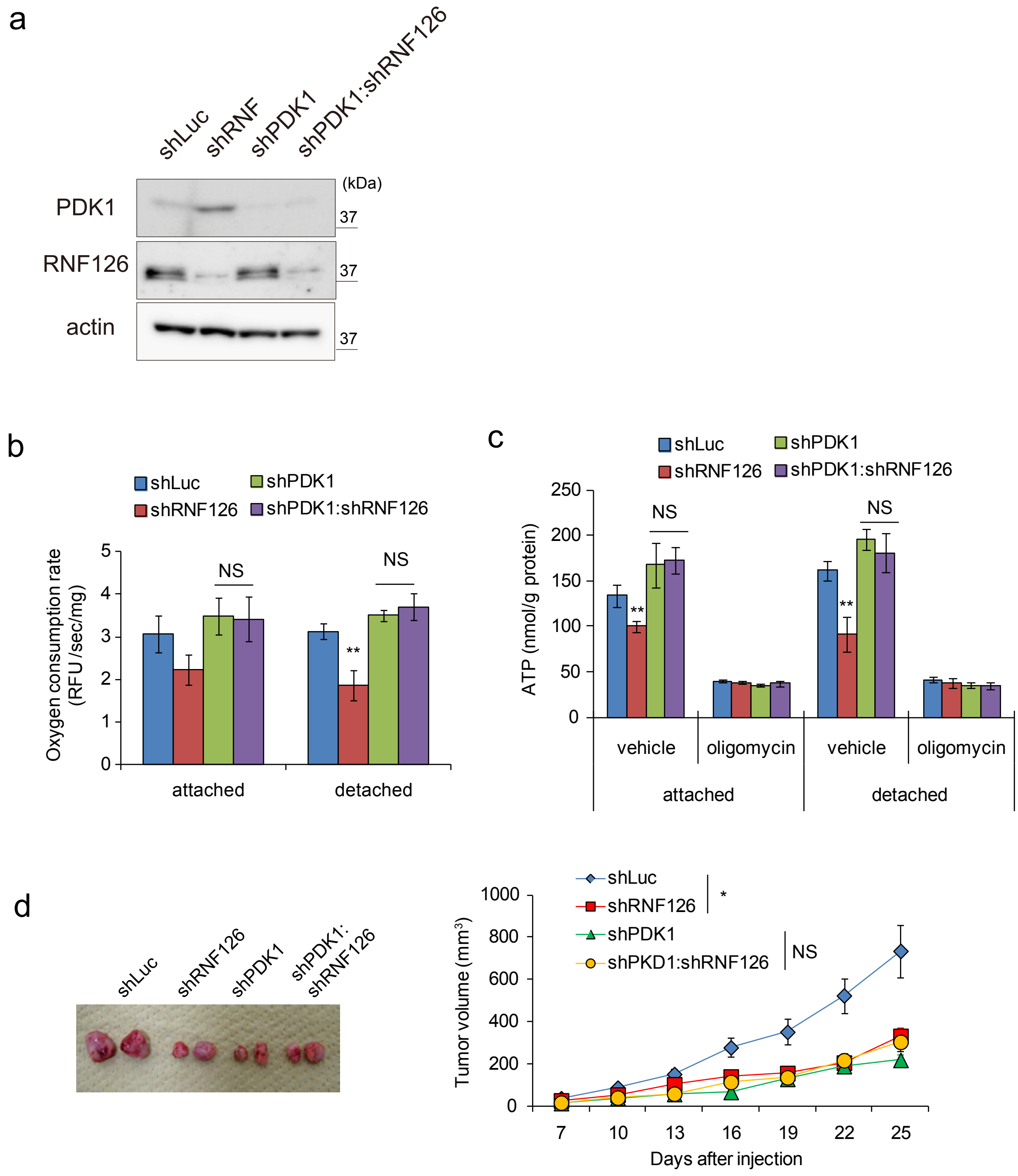

Figure S7 RNF126 regulates mitochondrial function and tumorigenicity in a PDK1-dependent manner in MDA-MB-231 cells.
(**a**) Western blot analysis of PDK1 and RNF126 in control, RNF126-, PDK1-, and RNF126 and PDK1-depleted MDA-MB-231 cells.
(**b**, **c**) Control, RNF126-, PDK1-, and RNF126 and PDK1-depleted MDA-MB-231 cells were cultured under attached or detached conditions, after which they were assayed to determine their oxygen-consumption rates (**b**) and cellular ATP contents (**c**).
(**d**) Tumor growth following subcutaneous implantation of control, RNF126-, PDK1-, and RNF126 and PDK1-depleted MDA-MB-231 cells in mice.
In **b** and **c**, the error bars indicate the standard deviations (n = 3); data were analyzed using the t-test. **p < 0.01. The data shown in **a**–**c** are representative of 3 independent experiments with similar results. In **d**, the error bars indicate the s.e.m.; n = 8 from 2 independent experiments (n = 4 and n = 4, respectively); the data were analyzed using the Mann–Whitney U-test. *p < 0.05, **p < 0.01.

Figure S8

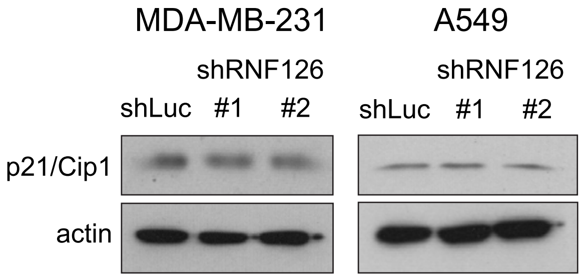

Figure S8 Knockdown of RNF126 has no apparent effect on p21/Cip1 protein level.
Western blot analysis of MDA-MB-231 and A549 cells following knockdown of RNF126. The data shown are representative of 3 independent experiments with similar results.

Figure S9

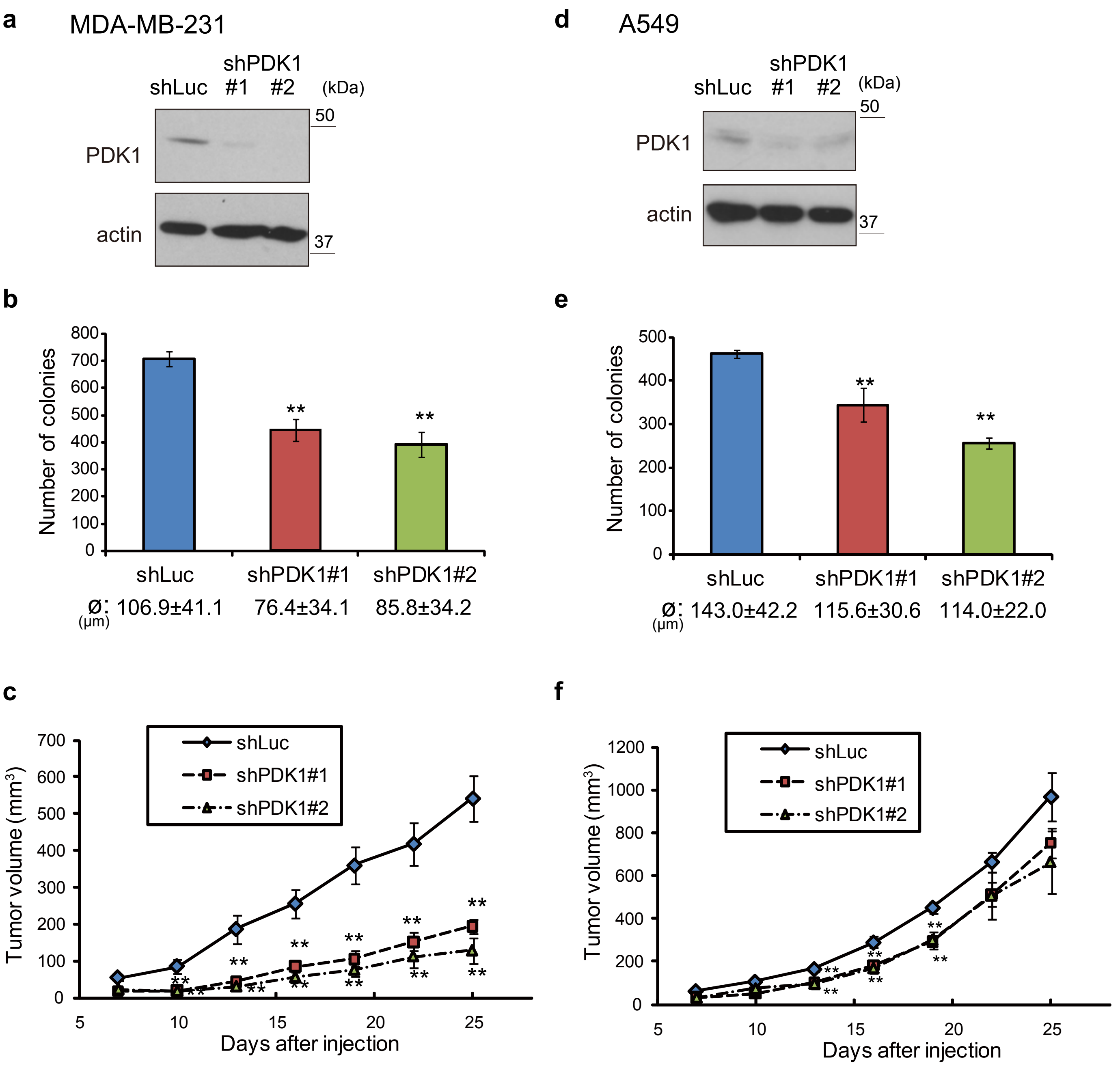

Figure S9 PDK1 depletion attenuates tumorigenicity.
(**a**, **d**) Western blot analysis of PDK1 expression in control and PDK1-depleted MDA-MB-231 cells (**a**) and A549 cells (**d**).
(**b**, **e**) Number of colonies formed in soft agar by control and PDK1-depleted MDA-MD-231 (**b**) and A549 cells (**e**). The diameter (ø) of the counted colonies is indicated as the average ± standard deviation.
(**c**, **f**) Tumor growth following subcutaneous implantation of control and PDK1-depleted MDA-MB-231 (**c**) and A549 cells (**f**) in mice. In **b** and **e**, error bars indicate the standard deviations (n = 3); data were analyzed using the t-test. **p < 0.01. The data shown are representative of 3 independent experiments with similar results. In **c** and **f**, the error bars indicate the s.e.m.; n = 8 from 2 independent experiments (n = 4 and n = 4, respectively); the data were analyzed using the Mann–Whitney U-test. *p < 0.05, **p < 0.01.

Figure S10

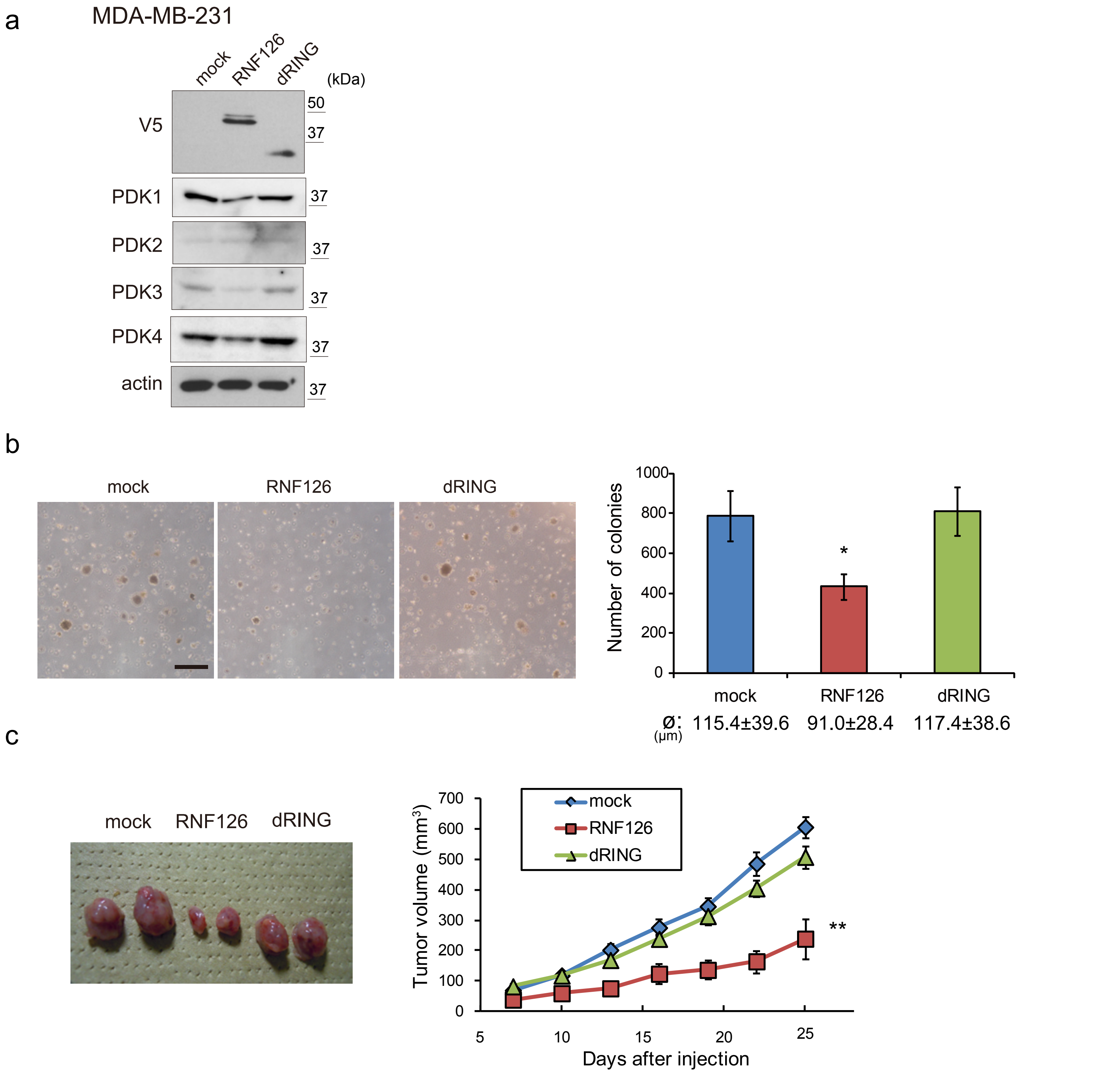

Figure S10 RNF126 overexpression attenuates tumorigenicity.
(**a**) Western blot analysis of PDKs in mock-treated MDA-MB-231 cells and MDA-MB-231 cells overexpressing V5-tagged wild-type RNF126 and the dRING mutant.
(**b**) (left) Representative photographs of colony formation in soft agar in mock, wild-type RNF126, and dRING mutant-expressing MDA-MD-231 cells. Scale bar = 500 μm. (right) Number of colonies formed in soft agar by mock, wild-type RNF126, and dRING-mutant expressing MDA-MD-231 cells. The diameter (ø) of the counted colonies is indicated as the average ± standard deviation.
(**c**) Tumor growth following subcutaneous implantation of mock, wild-type RNF126, and dRING mutant-expressing MDA-MB-231 cells in mice. (left) Representative photographs (day 25) and rate of growth (right).
In **B**, the error bars indicate the standard deviations. (n = 3); the data were analyzed using the t-test. **$p < 0.01$. The data shown in **a** and **b** are representative of 3 independent experiments with similar results. In **c**, the error bars indicate the s.e.m.; n = 8 from 2 independent experiments (n = 4 and n = 4, respectively); the data were analyzed using the Mann–Whitney U-test. *$p < 0.05$, **$p < 0.01$.

Figure S11

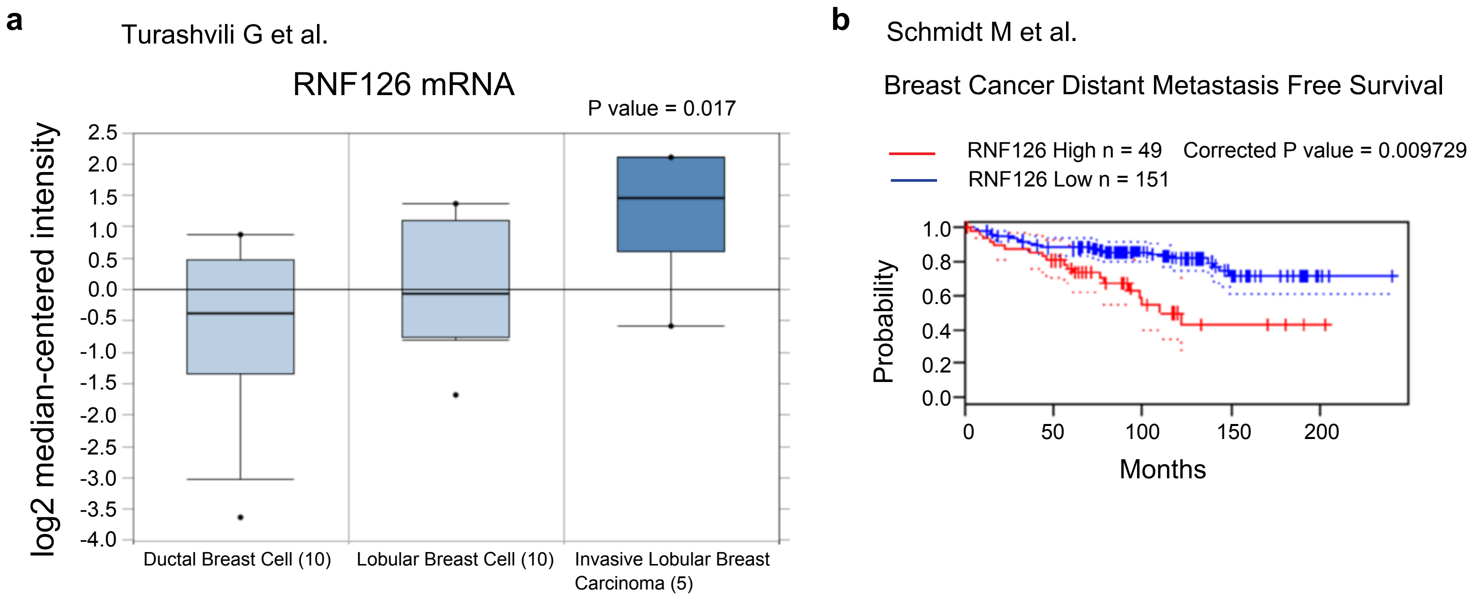

Figure S11 Database analysis of RNF126 expression in clinical samples.
(**a**) Expression of RNF126 in normal and breast cancer tissue was analyzed by the Oncomine database.
(**b**) Distant metastasis-free survival of RNF126-high and RNF126-low breast cancer patients was analyzed using the PrognoScan database.

Table S1.

Model ID	Model name	Score	Relative score	Start	End	Strand	predicted site sequence
MA0076.2	ELK4	5.834	0.831712180113285	-1964	-1954	1	GGTTTTCCTGG
MA0028.1	ELK1	6.479	0.827552294952313	-1961	-1952	-1	ATCCAGGAAA
MA0028.1	ELK1	6.017	0.812199429010522	-1915	-1906	1	GAGCAGGAAT
MA0028.1	ELK1	7.016	0.845397509261278	-1880	-1871	1	CGACTGGAAG
MA0028.1	ELK1	6.289	0.821238345755472	-1870	-1861	1	GAGGCTGAAG
MA0018.2	CREB1	7.861	0.864223403761055	-1754	-1747	-1	tgacctca
MA0018.2	CREB1	9.772	0.934207183734525	-1754	-1747	1	tgaggtca
MA0028.1	ELK1	6.527	0.829147397907304	-1618	-1609	1	aacccgggag
MA0028.1	ELK1	7.021	0.845563665819089	-1454	-1445	1	TTGCAGGAAG
MA0076.2	ELK4	11.789	0.924027619842049	-1452	-1442	-1	GGACTTCCTGC
MA0028.1	ELK1	7.273	0.853937956332794	-1390	-1381	1	ccctcggaag
MA0076.2	ELK4	4.802	0.81571393766155	-1388	-1378	-1	gggcttccgag
MA00	CREB1	6.296	0.806910679711	-1224	-1217	1	TGAggccg

18.2			615				
MA0018.2	CREB1	7.346	0.845363306070664	-1158	-1151	1	cgaggtca
MA0076.2	ELK4	4.103	0.804877918791625	-1010	-1000	-1	caacctccgcc
MA0018.2	CREB1	7.674	0.857375174114253	-894	-887	-1	TGAGGTTA
MA0076.2	ELK4	7.429	0.856438145918051	-820	-810	1	AGGTTTCCTGC
MA0028.1	ELK1	7.568	0.863741193243677	-817	-808	-1	AGGCAGGAAA
MA0018.2	CREB1	8.130	0.874074600418755	-739	-732	-1	TGAGGTCT
MA0018.2	CREB1	6.656	0.820094437320432	-722	-715	1	TGACCCCA
MA0028.1	ELK1	6.872	0.840612200396304	-634	-625	-1	GCGCCTGACA
MA0018.2	CREB1	7.206	0.840236289222791	-590	-583	-1	TCAGGTCA
MA0028.1	ELK1	7.133	0.849285572714069	-505	-496	1	gacctggaag
MA0076.2	ELK4	3.895	0.801653466824608	-406	-396	-1	gtacttcagcc
MA0076.2	ELK4	10.149	0.898604056255958	-284	-274	1	tctcttccgcc
MA0028.1	ELK1	7.910	0.875106301797991	-281	-272	-1	gcggcggaag
MA00	ELK1	10.595	0.964332373342	-254	-245	1	ccgccggaag

28.1			815				
MA0076.2	ELK4	13.304	0.947513411813346	-252	-242	-1	gggcttccggc
MA0076.2	ELK4	4.488	0.810846255365189	-145	-135	1	CCGCTCCCGCG

Table S1. **JASPAR motif analysis of RNF126 promoter.**

Supplementary information

Supplementary Materials and Methods

Trypan blue-exclusion assay

Cells (1×10^5) were cultured in standard or Ultra Low Cluster 6-well plates (Corning Inc.) for 96 h. Then, the cells were washed 3 times with PBS and incubated in Accumax Cell Dissociation Solution (Innovative Cell Technologies, Inc.**,** San Diego, CA, USA) for 15 min. Dissociated cells were stained with an equal volume of 0.4% trypan blue solution (Life Technologies), and trypan blue-positive cells were counted by microscopy.

/

Primers for real-time PCR

Following specific primers were used for real-time PCR: *PDK1* sense, 5′-tcctgtcaccagccagaatg-3′ and antisense, 5′-cttcctttgccttttccacc-3′; *PDK2* sense, 5′-tcctgtcaccagccagaatg-3′ and antisense, 5′-cttcctttgccttttccacc-3′; *PDK3* sense, 5′-tcctgtcaccagccagaatg-3′ and antisense, 5′-cttcctttgccttttccacc-3′; *PDK4* sense, 5′-tcctgtcaccagccagaatg-3′ and antisense, 5′-cttcctttgccttttccacc-3′.

Recombinant proteins

Recombinant proteins, such as GST and GST-RNF126, were expressed in *Escherichia coli*

BL21 Gold (DE3) pLysS (Stratagene, Santa Clara, CA, USA) using the pDEST15 expression vector (Invitrogen). Expression was induced at mid-log phase with 0.5 mM isopropyl-D-thiogalactoside and cells were incubated for a further 3 h at 37°C. Cells were harvested, then re-suspended in phosphate-buffered saline (PBS) containing 1% Triton X-100 and protease inhibitor cocktail III (Merck, Darmstadt, Germany), and sonicated. Following centrifugation, supernatants were applied to a glutathione–Sepharose 4B (GE Healthcare, Little Chalfont, UK) column and washed three times with PBS**.** Recombinant proteins were subsequently eluted in TBS buffer (50 mM Tris-HCl [pH7.6], 150 mM NaCl).

$(His)_6$-PDK1 recombinant protein was expressed in *Escherichia coli* BL21 Gold (DE3) pLysS (Stratagene) using the pDEST17 expression vector (Invitrogen). Induction of expression and preparation of cell lysates were performed as described above. Imidazole (10 mM) was added to the supernatant fractions, and samples were then loaded onto a Ni^{2+}-conjugated chelation sepharose column (GE Healthcare) and incubated for 1 h at 4°C. The beads were washed five times with 50 mM imidazole/TBS, and then the bound proteins were eluted with 500 mM imidazole and dialyzed against TBS. Protein purity was assessed by SDS-PAGE.

In vitro ubiquitination assay

In vitro ubiquitination assays were performed using an ubiquitinylation kit (Enzo Life Sciences,

Farmingdale, NY, USA) according to the manufacturer's instructions.

shRNAs

The shRNA sequences against *PDK1* were as follows: #1, 5′-GCUGAGGAUGCUAAAGCUAUUCGAAAAUAGCUUUAGCAUCCUCAGC-3′ and #2, 5′-GCAUAAAUCCAAACUGCAAUGCGAACAUUGCAGUUUGGAUUUAUGC-3′.

Antibody

Mouse anti-p21/Cip1 antibody was purchased from Cell Signaling Technology (Danvers, MA, USA).

Database analysis

For RNF126 expression analysis, we used the Oncomine database (https://www.oncomine.org/) and analyzed the GSE5764 dataset [1]. For metastasis-free survival analysis, we used the PrognoScan database (http://www.prognoscan.org/) and analyzed the GSE11121 dataset [2].

References

1 Turashvili G, Bouchal J, Baumforth K *et al.* Novel markers for differentiation of lobular and

ductal invasive breast carcinomas by laser microdissection and microarray analysis. *BMC cancer* 2007; **7**:55.

2 Schmidt M, Bohm D, von Torne C *et al.* The humoral immune system has a key prognostic impact in node-negative breast cancer. *Cancer Res* 2008; **68**:5405-5413.
